# Artificial intelligence applications in oxaliplatin-based chemotherapy for colon cancer: advancing prognosis, toxicity prediction, and dose personalization

**DOI:** 10.3389/fphar.2026.1784272

**Published:** 2026-08-10

**Authors:** Dana Jammoul, Felix Ajibuwa, Mostafa Jammoul, Sophie Schlosser-Hupf, Martina Müller, Nahed El-Najjar

**Affiliations:** 1 Department of Pharmacology and Toxicology, Faculty of Medicine, American University of Beirut, Beirut, Lebanon; 2 Department of Electrical and Computer Engineering, Maroun Semaan Faculty of Engineering and Architecture, American University of Beirut, Beirut, Lebanon; 3 Department of Internal Medicine I, Gastroenterology, Hepatology, Endocrinology, Rheumatology, and Infectious Diseases, University Hospital Regensburg, Regensburg, Germany

**Keywords:** artificial intelligence, chemotherapy-induced peripheral neuropathy, colorectal cancer, FOLFOX, oxaliplatin, precision medicine

## Abstract

Colorectal cancer (CRC) remains a significant cause of cancer deaths worldwide. Oxaliplatin-based regimens, such as FOLFOX, which form the cornerstone of therapy, are associated with variability in patient responses and toxicities, most notably peripheral neuropathy, and often limit long-term benefit. In recent years, the use of Artificial Intelligence (AI) in oncology has expanded significantly. The beneficial role of AI lies in its unprecedented ability to rapidly process and integrate high-dimensional datasets (e.g., genomic, radiomic, clinical) to uncover subtle, nonlinear relationships that conventional statistical methods cannot access. Thereby, AI is transforming the empirical approach to chemotherapy into a truly predictive science. Unlike previous reviews that broadly discuss AI in oncology, this review focuses specifically on oxaliplatin, drawing on genomic, transcriptomic, radiomic, and body composition data to refine patient stratification and anticipate potential adverse effects. It also highlights emerging AI-driven strategies for identifying transporter inhibitors and protective agents to mitigate neurotoxicity, particularly in patients with CRC. By moving beyond retrospective prediction, the review illustrates how AI can enable proactive, individualized treatment planning and safer dosing. It also summarizes and clarifies the methodologies used in machine learning models, serving as a reference for readers interested in this field. Collectively, the review highlights AI as a transformative tool for advancing precision oncology in oxaliplatin-based CRC care and provides the first comprehensive synthesis of AI applications in oxaliplatin therapy, with a focus on prognosis, toxicity prediction, and dose personalization.

## 1 Introduction

### 1.1 Colon cancer overview

**FIGURE 1:**
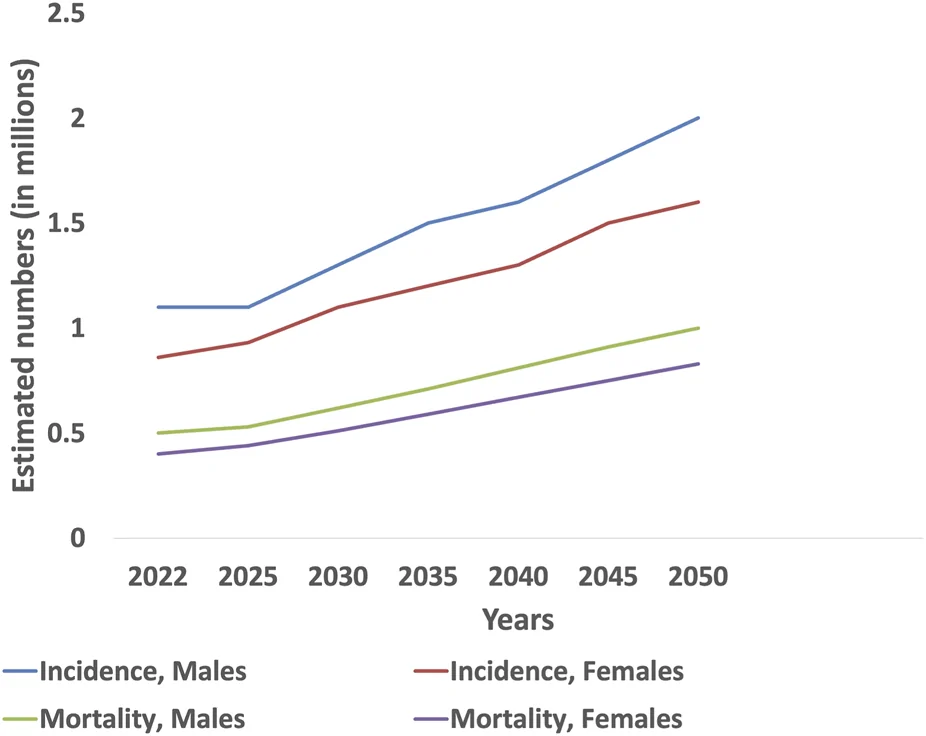
The graph illustrates the projected global incidence and mortality of colorectal cancer (CRC) from 2022 to 2050 among males and females (ages 0–85+). Solid lines represent incidence trends, while dashed lines indicate mortality trends. Estimates were generated using the Cancer Tomorrow projection tool of the International Agency for Research on Cancer (IARC) based on Globocan 2022 (version 1.1; data released 08 February 2024). © IARC/WHO 2025.

Colorectal cancer (CRC), a multifactorial disease influenced by both genetic and environmental factors, is a significant global health concern (Duan et al., 2022). Unfortunately, CRC remains the third most frequently diagnosed cancer worldwide and is the second leading cause of cancer-related deaths globally (Duan et al., 2022; Chen et al., 2024a). In 2020, approximately 1.9 million new CRC cases emerged, causing approximately 930,000 deaths worldwide (Organization, 2023). According to the Global Cancer Registry, there is an alarming and significant worldwide increase in the prediction of new incidence cases and deaths of both males and females by 2050 (Figure 1). Although CRC has traditionally been considered a disease of older adults (≥50 years), recent epidemiological data show a worrisome increase among younger individuals (Board, 2025). Notably, however, while early CRC tumors may be asymptomatic, making early diagnosis challenging, they are highly treatable when detected early; with localized disease often curable with endoscopy or surgery (Duan et al., 2022). The fact that surgery alone cures about half of patients with early-stage disease (Board, 2025) underscores the critical role of regular screening (e.g., colonoscopy) for early detection and improved outcomes; however, when diagnosed at later stages, survival rates from CRC decrease dramatically (Duan et al., 2022).

### 1.2 CRC treatment modalities

**FIGURE 2:**
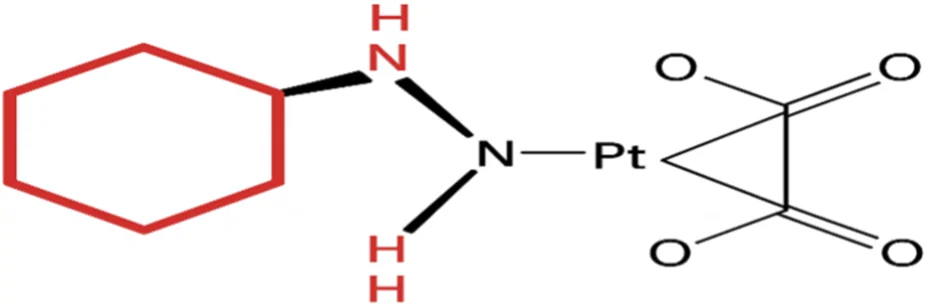
Chemical structure of oxaliplatin. The structure illustrates the unique DACH configuration (in red), responsible for its distinct pharmacological profile and reduced cross-resistance relative to cisplatin and carboplatin. Created in BioRender. El-Najjar, N. (2026) https://BioRender.com/87pnm4x.

CRC treatment depends on stage and patient factors, typically involving multiple treatment modalities (Duan et al., 2022), such as surgical resection (STAGES I-III) (Engstrom et al., 2003; Andre et al., 2015), radiotherapy (STAGES I-III) (Duan et al., 2022), chemotherapy (STAGE III) (Moore and Haller, 1999), targeted agents (e.g., anti-EGFR, anti-VEGF antibodies) (STAGE IV), and immunotherapy for selected advanced cases (STAGE IV) (Duan et al., 2022). In metastatic (STAGE IV) CRC, systemic chemotherapy (often with targeted drugs) may be administered pre- or post-surgery (Engstrom et al., 2003). Particularly, stage III patients routinely receive adjuvant chemotherapy, typically a fluoropyrimidine (5-fluorouracil [5-FU])-based regimen, to reduce recurrence risk (Moore and Haller, 1999). Oxaliplatin (Figure 2), a third-generation platinum-based chemotherapeutic agent, is particularly active against gastrointestinal (GI) cancers, especially as part of FOLFOX (5-FU + leucovorin [LV] + oxaliplatin) for metastatic CRC (Board, 2025). Of clinical relevance, the introduction of oxaliplatin into the FOLFOX regimen revolutionized CRC treatment, establishing it as the gold standard. Patients responding to oxaliplatin benefit from several aspects of the treatment. An analysis of 10-year survival data from the MOSAIC cohort showed that incorporating oxaliplatin into 5-FU/LV resulted in a 5.8% absolute increase in disease-free survival (DFS; hazard ratio [HR], 0.82 [95% CI, 0.71–0.95]; P = 0.007) as well as a 4.6% absolute increase in overall survival (OS; HR, 0.85 [95% CI, 0.73–0.99]; P = 0.043) (Andre et al., 2015). Similarly, the C-07 trial demonstrated a 5.2% absolute increase in 8-year DFS with oxaliplatin treatment (HR, 0.82 [95% CI, 0.72 to 0.93]; P = 0.002) (Yothers et al., 2011). The regimen is also standard for metastatic disease, often combined with targeted agents. CAPOX (capecitabine + oxaliplatin) is a widely accepted alternative (Board, 2025).

Notably, despite being one of the most commonly administered first-line chemotherapy regimens for patients with CRC, only about 50% of CRC patients exhibit positive responses after FOLFOX treatment (Colucci et al., 2005). Furthermore, oxaliplatin is associated with a high prevalence of both acute and chronic disabling peripheral neurotoxicity (Soveri et al., 2019). According to Kerckhove et al., more than 90% of patients receiving oxaliplatin therapy develop acute chemotherapy-induced peripheral neuropathy (CIPN), and 30%–50% of patients develop long-term CIPN (Kerckhove et al., 2017). Indeed, this showcases the importance of stratifying patients into responders and non-responders to oxaliplatin therapy before starting the chemotherapy regimen. This is especially crucial given that oxaliplatin-associated neuropathy typically emerges by the fourth or fifth month of treatment in most patients, and tumors acquire chemotherapy resistance over time, making the initial therapeutic impact particularly important (Abraham et al., 2021a).

### 1.3 Mechanism of action of oxaliplatin and predictors of response variability

The cytotoxicity of oxaliplatin stems from its platinum moiety, which covalently binds to guanine bases in DNA, forming cross-links that stall replication forks (Meyerhardt and Mayer, 2005; Alcindor and Beauger, 2011). Oxaliplatin’s alkylating effect is not cell-cycle-specific, allowing it to kill both dividing and non-dividing tumor cells (Duan et al., 2022). Yet, oxaliplatin is particularly effective against rapidly proliferating GI tumor cells (Alcindor and Beauger, 2011). Following administration, oxaliplatin is quickly metabolized, with approximately half of the platinum excreted in the urine (Devanabanda and Kasi, 2025). Several factors, such as differences in oxaliplatin absorption, metabolism, and DNA repair mechanisms among individual patients, can alter the extent of oxaliplatin accumulation in both tumor and normal tissue DNA, thus impacting the drug’s effectiveness and associated toxicities (Wang et al., 2016). Different oxaliplatin response markers have been described in the literature, including those related to oncogene mutations (such as KRAS) (Shimizu and Nakayama, 2020), excision repair protein levels (such as ERCC-1) (Farina et al., 2022), and microsatellite instability (Cohen et al., 2021). Recent advances in molecular data generation, together with progress in artificial intelligence (AI), now offer powerful opportunities to elucidate resistance mechanisms, predict treatment response, and develop personalized strategies to improve clinical outcomes (Farina et al., 2022).

### 1.4 Artificial intelligence in oncology

**FIGURE 3:**
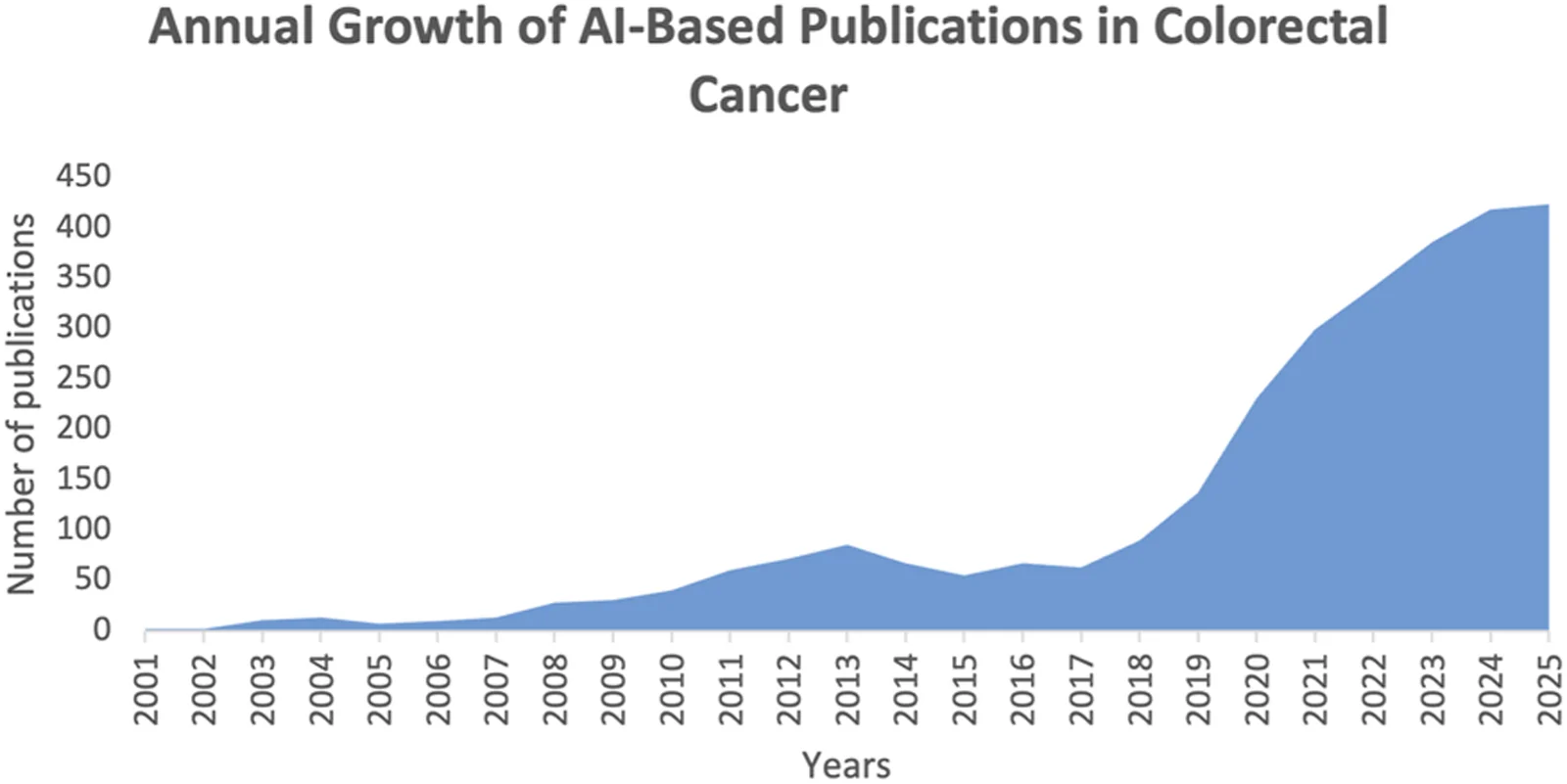
Overview of yearly trends in AI-driven research in CRC. Data extracted from a PubMed search covering the last 25 years and performed using the following MeSH query: (“Artificial Intelligence”[Mesh] OR “Machine Learning”[Mesh] OR “Deep Learning”[Mesh]) AND “Colorectal Neoplasms”[Mesh].

The role of AI in the healthcare system, in particular oncology, is well established. AI has been reliable in radiology (tumor detection), pathology (histology slide analysis), genomics (inferring mutational status), and electronic health records (outcomes prediction) (Shimizu and Nakayama, 2020). AI’s ability to process multidimensional data for pattern recognition, prognostic scoring, and decision support is key to its utility in cancer care (Shimizu and Nakayama, 2020; Farina et al., 2022). The increasing number of Food and Drug Administration (FDA)-approved AI diagnostic tools highlights the accelerating role of AI in medicine (Shimizu and Nakayama, 2020). Interestingly, specifically regarding CRC, Figure 3 shows a notable annual increase in AI-driven publications, with a steep acceleration starting around 2019. This trend highlights the growing interest in leveraging AI (Moore and Haller, 1999) and machine learning (ML) to address the diagnostic, prognostic, and therapeutic challenges posed by CRC.

Building on recent advances in computing power, data availability, and AI-driven analytics, this review addresses a gap in the existing CRC literature, which has largely focused on surgical applications and general prognosis. We aim to synthesize current evidence on how AI can be specifically leveraged to improve prognosis, predict response, and anticipate toxicity in oxaliplatin-based CRC therapy, thereby outlining new opportunities to enhance clinical outcomes in this patient population.

## 2 AI methodology in oncology

Machine learning (ML) is a computer-based methodology that learns from data and generates predictions without being explicitly programmed (Sil et al., 2019; Touretzky et al., 2023; Zaresefat and Derakhshani, 2023). ML models improve as more data become available and can incorporate complex information such as patient records, laboratory results, and medical images (An et al., 2023). ML key subtypes include: (a) Supervised Learning, which uses datasets with labeled outcomes, such as confirmed diagnoses. Here, techniques such as Support Vector Machine (SVM) and Random Forest (RF) are commonly utilized (Furizal et al., 2023; Saputra et al., 2021). This latter one is especially valued for its robustness in predicting adverse drug reactions (Hu et al., 2024); (b) Unsupervised Learning (UL) detects patterns in unlabeled data, for example, grouping patients with shared but non-obvious features. UL standard methods include K-Means and Hierarchical Clustering (Furizal et al., 2023); and (c) Reinforcement Learning (RL), which involves a decision-making “agent” that learns through trial and error, making it useful for personalized care that adapts over time (Furizal et al., 2023; Banumathi et al., 2025). Deep Learning (DL), a branch of ML, uses neural networks to process raw data and identify higher-level patterns (Hu et al., 2019; Park et al., 2020). To date, DL has driven significant advances in the healthcare system (Lotter et al., 2024). DL main subtypes include: (a) Convolutional Neural Networks (CNNs): Designed for image-based data, whereby it excels in tasks such as classification, tumor detection, and scan segmentation, at times exceeding human accuracy (Feng and Tang, 2023; Mienye et al., 2025; Taye, 2023); (b) Recurrent Neural Networks (RNNs) and Transformers: Suitable for sequential data, such as medical records. Transformers, a newer design, manage long-range data more effectively than RNNs; hence, they are used in cardiotoxicity prediction (Shickel et al., 2017; Kim, 2025); (c) Natural Language Processing (NLP): Focuses on interpreting unstructured text, including clinical notes and reports (Park et al., 2020; Abbasgholizadeh Rahimi et al., 2021; Jerfy et al., 2024). NLP supports applications such as data extraction, transcription, and the identification of disease trends (Jerfy et al., 2024). A combination of methods is sometimes used to enhance applicability. For instance, it has been demonstrated that combined methods, such as Depp RL, which combines RL (precision dosing) with neural networks (DL), are more effective at interpreting complex inputs, such as imaging (Banumathi et al., 2025; De Giorgi et al., 2022; Ribba, 2023).

**FIGURE 4:**
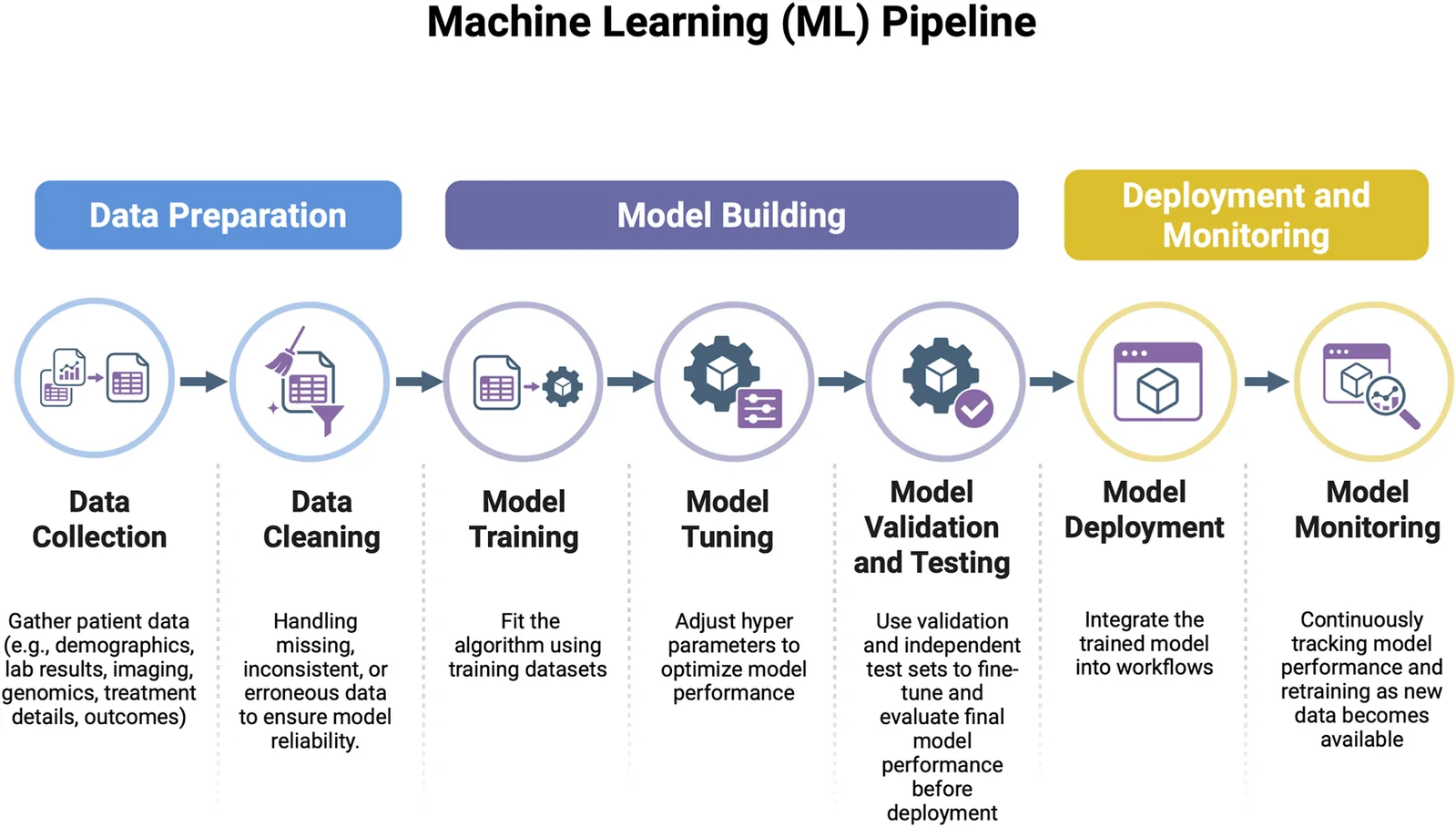
Overview of the Machine Learning (ML) pipeline. The workflow illustrates the main stages of ML model development, including data preparation (collection and cleaning), model building (training, tuning, validation, and testing), and model deployment and monitoring. Created in BioRender. El-Najjar, N. (2026) https://BioRender.com/zxeyrhl.

Developing an ML model capable of transforming raw biomedical data into predictive clinical tools involves a series of steps that form the foundation of the ML pipeline, collectively driving AI innovation in healthcare and oncology. Figure 4 illustrates the interconnected stages of data preparation, model building, and deployment. Briefly, the process begins with data acquisition and preprocessing, where clinical, molecular, and imaging data are curated and cleaned to ensure quality. This is followed by feature extraction and selection, which identify the most relevant variables contributing to prediction accuracy. Next, different algorithms are trained and validated, allowing models to learn from data patterns while avoiding overfitting. Finally, the optimized model is tested and deployed for clinical application, with continuous performance monitoring to ensure reliability and generalizability across patient populations.

### 2.1 Artificial intelligence for predicting response to oxaliplatin-based chemotherapy in colon cancer

Predicting patient response to cancer treatment remains a major unmet need in both clinical practice and drug development (Zhang et al., 2022). Accurate response prediction could inform the design of more effective therapies and enable individualized treatment protocols that maximize efficacy while minimizing unnecessary toxicity (Hartl et al., 2021).

One of the leading causes of treatment failure in cancer patients is drug resistance, resulting from numerous factors, such as tumor growth kinetics, tumor burden, tumor heterogeneity, tumor microenvironment (TME), physical barriers, the immune system, the undruggable genome, and therapeutic pressures (Vasan et al., 2019; Zhu et al., 2021). In fact, the effectiveness of a drug is primarily determined via three key components: the therapeutic targets, the cancer cells themselves, and TME (Zhang et al., 2022). Conventional tumor type-dependent anticancer treatments such as chemotherapy frequently produce suboptimal outcomes in addition to significant side effects; as a result, they are widely seen as one-size-fits-all approaches (Hartl et al., 2021).

Evaluating drug response through clinical trials is challenging due to high costs and the time-intensive nature of the experiments. Consequently, AI has attracted significant attention for overcoming these challenges. In recent years, AI has dramatically advanced the prediction of drug response. Large-scale datasets of drug response for cell lines, such as Cancer Cell Line Encyclopedia (CCLE), Genomics of Drug Sensitivity in Cancer (GDSC), Cancer Therapeutics Response Portal (CTRP), and Genentech Cell Line Screening Initiative (gCSI), are established, enabling the use of ML and AI techniques to predict drug response computationally (Lu et al., 2020).

**FIGURE 5:**
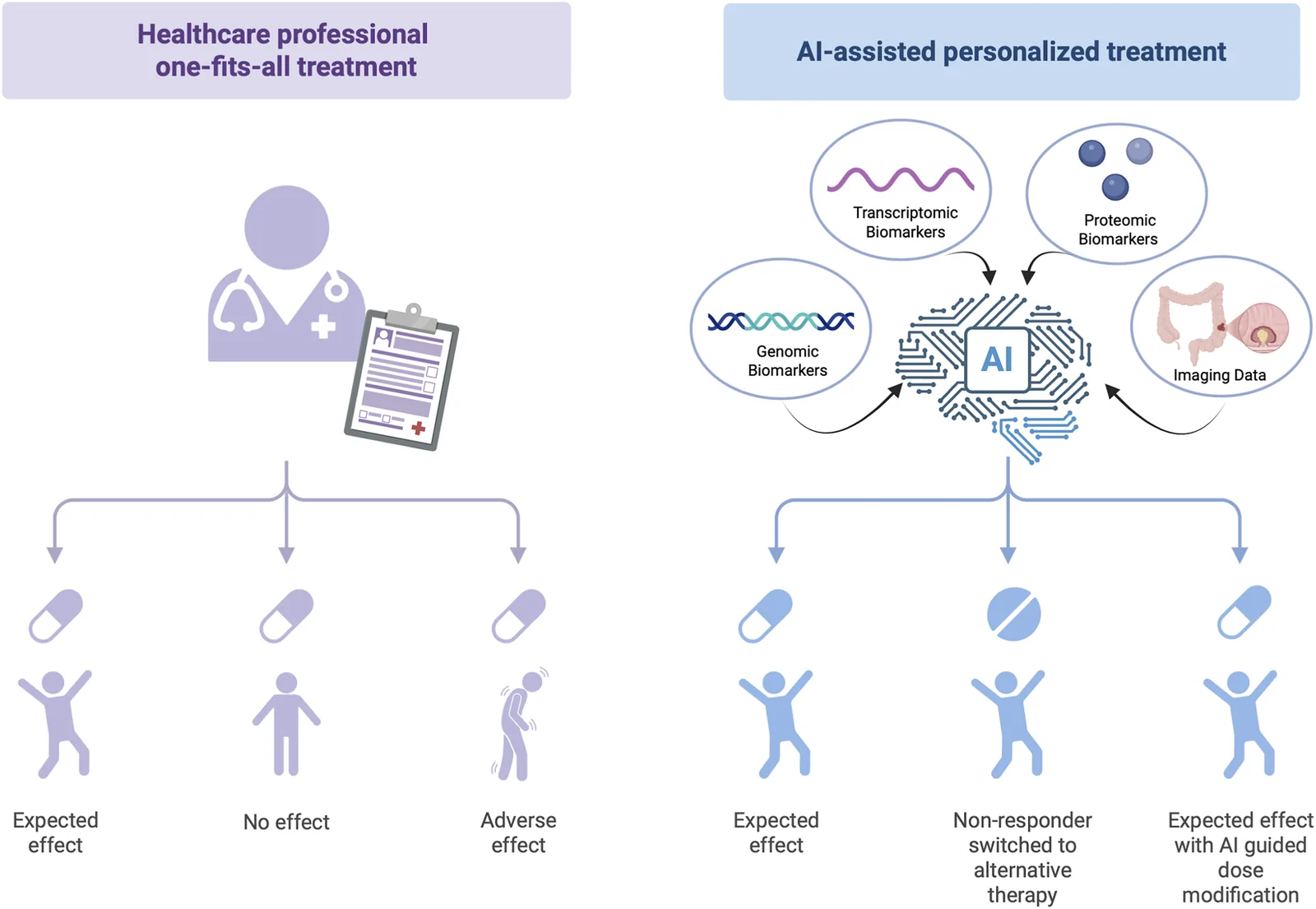
AI-driven precision medicine approach for colorectal cancer management. Created in BioRender. El-Najjar, N. (2026) https://BioRender.com/xbv4cfs.

Personalizing therapy requires incorporating several parameters that can influence treatment response. As illustrated in Figure 5, numerous data inputs can be integrated to train AI models to predict clinical outcomes in patients with CRC. For example, genomic and transcriptomic features can identify molecular signatures linked to oxaliplatin-based chemotherapy sensitivity and resistance. Moreover, proteomic data can further reveal dysregulated protein pathways associated with drug response. Lastly, radiomic features extracted from medical images provide non-invasive biomarkers that reflect the tumor phenotype and microenvironment. Therefore, by combining multimodal features, the ML model can identify responders and personalize oxaliplatin-based chemotherapy dosing. A detailed analysis of the current knowledge on each of the factors mentioned above that influence response to treatment is provided below.

### 2.2 Genomic biomarker-based predictions

Genomic biomarkers play a crucial role in enabling AI applications to accurately predict response to anticancer therapies. Several approaches were used to identify genes associated with treatment response. For example, Amniouel et al., identified gene signatures capable of predicting patient response to FOLFOX and FOLFIRI in both early-stage and metastatic CRC using a newly developed ML model. At the early stages of CRC, 10 genes (FOXA1, KRT23, GRM8, HOXA11, HOXA10, ABCB1, LEFTY1, CHRM3, OLMF4, and LYZ) were identified as relevant to drug response prediction. Interestingly, in metastatic CRC, 12 other genes (IFI44L, WIF1, LY6G6D, SFRP2, RETNLB, CMPK2, ACSL6, RSAD2, LOC100507477, IFIT1, MX1, and HUNK) were associated with drug response prediction (Amniouel and Jafri, 2023). The differences observed in the genes used to distinguish responders from non-responders to FOLFOX across all stages of CRC align with the differential gene expression profiles between early-stage and metastatic CRC (Poturnajova et al., 2021; Peixoto et al., 2023). Additionally, the genes that distinguished responders from non-responders to FOLFOX were different from those that did so for FOLFIRI, except for one gene present in both: secreted frizzled-related protein 2 (SFRP2). This suggests that patients who do not respond to FOLFOX may respond to FOLFIRI, and *vice versa*. In fact, the analysis of Amniouel et al. estimated that on average, 28.6% of patients who failed one drug treatment regimen would have likely responded to the other treatment regimen. It is worth noting that, among the ML models tested in the study, RF models consistently outperformed SVM models (Amniouel and Jafri, 2023). This finding aligns with the existing literature, which indicates that RF handles noisy data and outliers better than SVM (Sabzekar et al., 2021; Wang and Li, 2017). In another study, Lu et al. performed microarray meta-analysis to identify differentially expressed genes (DEGs) between FOLFOX responders and non-responders in metastatic or recurrent CRC patients. Interestingly, five genes (WASHC4, HELZ, ERN1, RPS6KB1, and APPBP2) were downregulated in FOLFOX responders, while 13 genes (IRF7, EML3, LYPLA2, DRAP1, RNH1, PKP3, TSPAN17, LSS, MLKL, PPP1R7, GCDH, C19ORF24, and CCDC124) were upregulated in FOLFOX responders. The 18 modulated genes were then used on several ML algorithms to predict response to FOLFOX. The SVM algorithm achieved the highest performance, with a sensitivity of 0.900 (95% CI: 0.669–0.982) and a specificity of 0.692 (95% CI: 0.389–0.896). The RF algorithm yielded comparable results, with a sensitivity of 0.850 (95% CI: 0.611–0.960) and the same specificity (Lu et al., 2020; Statnikov et al., 2008). Among the top 18 gene panels, high expression levels of MLKL and CCDC124 (both of which are upregulated genes in FOLFOX responders) were significantly associated with improved overall survival in metastatic CRC patients undergoing FOLFOX treatment (Lu et al., 2020). Although CCDC124 has not been linked to antineoplastic drug resistance, MLKL plays a key role in TNF-induced necroptosis and mediates antimicrobial peptide HPA3P-induced necrotic death in colon cancer (Cho et al., 2018). In fact, Sun et al. developed a nanoscale cationic liposome system that encapsulates MLKL-pDNA, a SMAC mimetic, and zVAD to overcome multidrug resistance in colon cancer cells. Therefore, MLKL may serve as a promising therapeutic target for FOLFOX-resistant metastatic CRC (Sun et al., 2018). Moreover, Tsuji et al. employed the RF algorithm to identify classifier genes that predict response to FOLFOX therapy in 83 patients with unresectable CRC receiving FOLFOX treatment. All CRC samples were obtained before initiation of mFOLFOX6 therapy (Irigoyen et al., 2010), and after four cycles of mFOLFOX6, CT scans were performed, and gene expression was analyzed. CT scans were used to classify lesions as complete response (CR), partial response (PR), stable disease (SD), or progressive disease (PD) based on RECIST criteria and changes in tumor size (Therasse et al., 2000). The RF algorithm was then applied to the training set, yielding 1,197 informative probe sets that distinguish responders from non-responders to FOLFOX therapy. If multiple probes corresponding to the same gene were present, only one representative probe was selected. The out-of-bag classification accuracy was maximized when the top 15 probes [14 genes (SMURF2, MBTD1, AP3M2, RNF141, NPEPPS, BPTF, FAM73A, APPBP2, AMZ2P1, SRGAP1, NMT1, CSPP1, EIF1, and CEP290)] were used in the RF analysis. Using these genes, the RF model accurately identified 21 out of 23 responders (91.3% sensitivity) and 22 out of 23 non-responders (95.6% specificity), achieving an out-of-bag classification accuracy of 80.2%. The 14 predictor genes showed higher expression in non-responders, and samples with strong upregulation of these genes were more confidently predicted to be non-responders. Among the predictor genes identified by Tsuji et al., several were found to be associated with cisplatin response. An example is SMURF2, a member of the HECT family of E3 ubiquitin ligases, which likely regulates Smad2 expression by ubiquitinating and promoting proteasome-dependent degradation during TGFβ signaling (Lin et al., 2000; Zhang et al., 2001). Also, some studies have shown that TGFβ enhances cancer cell sensitivity to cisplatin; therefore, SMURF2 (which influences TGFβ signaling) serves as a biomarker for identifying likely responders to FOLFOX therapy (Thavaraj et al., 2005; Irigoyen et al., 2010). Notably, the ANKRD40 mRNA transcript was elevated in non-responders to FOLFOX. This finding aligns with previous research indicating that ankyrin-repeat proteins, such as p16 (Wei et al., 2021) and Notch proteins (Kim et al., 2019), have been linked to cancer. Thus, ankyrin-repeat domains may enhance platinum resistance. Mucaki et al. applied biochemically inspired ML, which uses established biochemical mechanisms to guide model design, to predict and compare cellular and patient responses to cisplatin, carboplatin, and oxaliplatin. Data were collected from breast and bladder cancer cell lines with measured GI_50_/IC_50_ values, in addition to RNA-seq gene expression and clinical outcome data from cisplatin-treated bladder, carboplatin-treated ovarian, and oxaliplatin-treated CRC patients in The Cancer Genome Atlas (TCGA) and Gene Expression Omnibus (GEO). Using gene expression signatures, chemotherapy response was predicted by GI_50_ threshold-dependent and threshold-independent ML models. In the GI_50_ threshold independent model, a set of candidate gene signatures was generated by shifting the GI_50_ thresholds that distinguish sensitivity from resistance. This was important to assess whether genes influencing drug responses remain consistent across different cell lines, since each cell line has different GI_50_ values. In fact, the overall distribution of genes across GI_50_ thresholds showed numerous clear similarities to the gene signatures identified by misclassification. Data from TCGA patients with CRC were used to test the oxaliplatin signatures. For oxaliplatin, the ensemble ML model (mean = −0.12 ± 2.7 HU) demonstrated the highest accuracy after 1 year, achieving 60% accuracy in disease-free patients and 73% in recurrent patients. The best-performing gene signature (designated as Oxa1) was: BRAF, FCGR2A, IGF1, MSH2, NAGK, NFE2L2, NQO1, PANK3, SLC47A1, SLCO1B1, UGT1A1. Oxa1 was more effective at correctly identifying disease-free patients than patients with recurrent disease at the 1-year treatment threshold. Interestingly, removing MSH2 from any gene signature in which it was included led to a significant increase in misclassification rates. For example, the misclassification of Cis14 (a gene signature for bladder cancer) and Oxa21 increased by 28.2% and 19.1%, respectively (Mucaki et al., 2019).

Interestingly, Abraham et al. validated FOLFOXai, a molecular signature predictive of treatment benefit from FOLFOX, developed by Caris Life Sciences. The study involved analyzing an integrated dataset containing comprehensive molecular profiling and clinical outcomes data. The clinical validation followed a two-step approach: first, synthetic study cohorts were assembled from real-world evidence (RWE) data; then, a retrospective-prospective analysis of samples from the TRIBE2 study was conducted. The FOLFOXai signature identified patients in both oxaliplatin-based arms of the randomized TRIBE2 trial who would ultimately have improved benefit (IB), with a clinically relevant increase in OS of 6 months in the FOLFOX/bevacizumab arm and 14.9 months in the FOLFOXIRI/bevacizumab arm. Additionally, the 67 genes identified by the final model provide novel insights into potential biological mechanisms underlying intrinsic resistance to FOLFOX/bevacizumab or the FOLFOX combination. Among those are genes involved in several critical pathways such as: WNT signaling (BCL9 and CDX2), epithelial to-mesenchymal transition (EMT) (INHBA, PRRX1, PBX1, and YWHAE), chromatin remodeling (EP300, ARID1A, SMARC4, and NSD3), DNA repair (WRN and BRIP1), NOTCH signaling (MAML2), and cell-cycle regulation (CNTRL and CCNE1) (Abraham et al., 2021a). Interestingly, both WNT signaling factors and EMT mediators are well-known contributors to chemotherapy resistance (Conroy et al., 2011). Moreover, BCL9, a transcriptional coactivator in the canonical WNT pathway and shown to promote a stem cell-like phenotype (Wang et al., 2019; Zhou et al., 2023), has been linked to platinum resistance in non-small cell lung cancer (Wang et al., 2014). To date, the Consensus Molecular Subtype (CMS) classification is the most widely recognized system, categorizing CRC into four CMS categories (CMS1-4), each associated with distinct prognostic outcomes (Guinney et al., 2015). These outcomes range from favorable survival in CMS2 to poor relapse-associated survival in CMS1, with CMS4 consistently having the worst overall prognosis (Guinney et al., 2015). Interestingly, with FOLFOXai, cancers predicted to have IB from FOLFOX/bevacizumab were more commonly associated with the CMS2 subtype; however, cancers classified as CMS1 were more frequently expected to exhibit decreased benefit (DB) from the same treatment (Abraham et al., 2021a).

It is noteworthy that previous prediction methods often treat the gene expression of all FOLFOX non-responders among CRC patients as a uniform group. However, an effective method should begin by dividing FOLFOX non-responders into specific subgroups based on common patterns of resistance. Then, each subgroup of FOLFOX non-responders should be compared individually with FOLFOX responders to analyze their particular resistance pathways. Thus, Tian et al. developed an iterative supervised learning (IML) method to identify the major subgroups of CRC patients who share a common mechanism of resistance to FOLFOX treatment. The IML method was validated in two independent datasets, one for FOLFOX-treated stage IV patients and the other for FOLFOX-treated stage III patients. In FOLFOX-treated stage IV CRC, the predicted FOLFOX responder group exhibited a 3-year survival rate of 61.9% (95% CI: 41.9%–91.4%), whereas the predicted FOLFOX non-responder group had a 3-year survival rate of 18.8% (95% CI: 6.8%–52.0%). To evaluate the predictive performance of the IML model in the adjuvant setting, the cohort of stage III CRC patients who had received FOLFOX as adjuvant therapy was applied to the model. Within the mentioned cohort, the focus was specifically on CMS4 patients, who have the worst prognosis, a higher risk of metastasis, and a larger sample size than stage III patients. The findings of this study suggest that there are at least two distinct major patterns of resistance to FOLFOX therapy. The dominant pattern of FOLFOX resistance, denoted as the IML1 group (representing approximately 75% of the non-responders), involves a synergistic combination of enhanced DNA damage repair, resistance to apoptosis, and altered tumor cell cycle regulation. The second pattern of FOLFOX resistance, classified as IML2, is characterized by BRCA1 activation and accounts for approximately 25% of non-responders. Importantly, these two patterns of FOLFOX resistance are mutually exclusive, suggesting they may have evolved independently. In terms of treatment selection, CRC patients in the IML1 subgroup of FOLFOX non-responders exhibit little BRCA1 activation; therefore, they may be more susceptible to PARP inhibitors, such as Olaparib, than those in the IML2 subgroup. In addition, among stage III CMS4 patients, the IML identified a subgroup with an epithelial-mesenchymal subtype who still responded to FOLFOX. This suggests that IML adds predictive value, whereas EMT alone does not always predict FOLFOX resistance (Tian et al., 2021).

### 2.3 Transcriptomic biomarker-based predictions

Based on the fundamental assumption that transcriptomic patterns reflecting the cellular states of cancer cells provide insight into drug responses, Chen et al. developed a new ML model, the colon oxaliplatin signature (COLOXIS). This model can predict the efficacy of oxaliplatin-based regimens using tumor transcriptomes as input features. The model classified patients into two distinct subpopulations: predicted responders (labeled as COLOXIS +) and predicted non-responders (labeled as COLOXIS -). The analysis assessed whether: 1) the model could predict patient outcomes based on recurrence-free survival (RFS) with 5-FU/LV or FOLFOX treatment, and 2) the COLOXIS model could predict oxaliplatin benefit by comparing treatment effects between the COLOXIS+ and COLOXIS–groups. The COLOXIS + group showed a significant benefit from oxaliplatin treatment (HR, 0.63 [95% CI, 0.46–0.86]; P = 0.004), with RFS of 79.7% in patients treated with FOLFOX compared with 69.3% in those treated with 5-FU/LV. However, the addition of oxaliplatin did not improve OS in the COLOXIS group (HR, 0.998 [95% CI, 0.72–1.39]; P = 0.99). As such, those patients may require alternative therapies to improve their outcomes. The COLOXIS model stratified patients into two nearly equal groups; accordingly, if COLOXIS predictions guided adjuvant treatment decisions, approximately half of CRC patients (those classified as COLOXIS−) could potentially be spared unnecessary treatment-related toxicity (Chen et al., 2024a).

Long non-coding RNAs (lncRNAs) have emerged as promising candidates for therapeutic intervention and prognosis prediction (Zhang Y. et al., 2022). In fact, multiple lncRNAs have been linked to oxaliplatin sensitivity in cancer. For instance, silenced LINC01134 enhances oxaliplatin sensitivity in hepatocellular carcinoma (Kang et al., 2022), LINC00963 influences oxaliplatin’s suppressive effect in gastric cancer by targeting ATG16L1 expression (Hou et al., 2021), and knocking down LINC00525 increases CRC sensitivity to oxaliplatin (Wang et al., 2019). Based on this, Zhou et al. screened lncRNAs associated with oxaliplatin sensitivity across multiple tumor cell lines and constructed a predictive model using an ML algorithm. The tumor cells that were sensitive to oxaliplatin were those from acute lymphoblastic leukemia (ALL), CRC, and acute myeloid leukemia (AML). 13 LncRNAs associated with oxaliplatin sensitivity, identified in these cells, through differential expression analysis, were used to construct a predictive model for assessing oxaliplatin sensitivity in tumor cells. The lncRNAs were incorporated into four ML algorithms: LASSO, Decision Tree (DT), RF, and SVM. The ML algorithms identified seven significant lncRNAs in the model, which demonstrated high predictive value for CRC patients undergoing oxaliplatin-based chemotherapy. However, Zhou et al.’s validation analysis failed to confirm the expression of all lncRNAs but was able to identify four consistent lncRNAs (C20orf197, UCA1, MIR17HG, and MIR22HG) (Zhou et al., 2023). Notably, among the four lncRNAs, only UCA1 has been linked to oxaliplatin sensitivity in both hepatocellular carcinoma (Wang et al., 2021) and CRC (Sun et al., 2019). As such, further investigation is needed to determine the roles of the remaining three lncRNAs and their association with oxaliplatin sensitivity.

### 2.4 Predictions of chemotherapy response based on proteomic biomarkers

Growing evidence suggests that some cytokines and their receptors play a key role in regulating the TME and contribute to many diseases, ranging from inflammatory bowel disease to carcinogenic processes. Some host-derived cytokines help suppress tumor formation by modulating immune responses (Balkwill, 2004) (Benelli et al., 2006; Seruga et al., 2008); conversely, others promote tumor growth, invasion, and metastasis by participating in various carcinogenesis-related processes, such as inflammation and uncontrolled growth signaling (Mantovani et al., 2008). Based on that, Chen et al. developed a novel predictive cytokine-based prognostic classifier (CBPC) to improve the prediction of patient outcomes. In this study, a multiplex bead-based Luminex assay was used to simultaneously detect 39 circulating cytokines in serum samples from metastatic CRC patients collected before they began first-line chemotherapy (Chen et al., 2015). SVM methods and Cox proportional hazards models were employed to construct the prognostic classifiers. Of the 39 cytokines, 17 were identified as significant prognostic factors in both the training and validation cohorts. In the combined cohort, the median OS was 18.0 months for the high-risk patients and 56.0 months for the low-risk patients. The CBPC was also strongly predictive of OS when all patients were analyzed together as a single cohort. Patients in low-risk groups generally responded favorably to their initial chemotherapy. Multivariate analyses across all study cohorts showed that serum levels of MDC, Flt3L, MCP-3, IL-8, IP-10, and MCP-1 correlated with OS. In fact, accumulating evidence has shown that IL-8 plays a key role in tumor progression, associated with metastasis in nasopharyngeal carcinoma (Li et al., 2012) and linked with shorter PFS in CRC patients undergoing chemotherapy (Ning and Lenz, 2012). This data further confirms the strong link between IL-8 and CRC progression, suggesting that a CBPC panel of 17 circulating cytokines can improve the prognosis prediction of CRC patients.

### 2.5 Radiomics and imaging-based predictions

Medical imaging offers a unique, highly accessible approach to obtaining tumor-related data compared with tissue sequencing. It enables the macroscopic assessment of tumor cells, the microenvironment, and adjacent tissue at the voxel level (Gillies et al., 2016). Radiomics is a rapidly growing research area that transforms standard patient imaging into extractable quantitative data (Aerts et al., 2014). Since its introduction in 2012, radiomics has been widely used in cancer research, demonstrating promise as a non-invasive, efficient approach for diagnosing, classifying, and predicting patient outcomes in oncology (Moore and Haller, 1999). The radiomics pipeline involves various key stages, including: 1) the collection of high-quality, multi-dimensional data, including medical images and clinical information from multiple centers; and 2) a pre-processing step (co-registration, noise reduction, and intensity normalization) to enhance image quality and standardize images across different scanners and protocols (van Timmeren et al., 2020). Defeudis et al. developed and validated an ML algorithm to predict the response of liver metastases using CT scans. The study compared radiomics results from two feature-extraction approaches: a 3D approach, in which features are extracted from the entire metastasis, and a region-of-interest (ROI) approach, which extracts features from a 7 × 7 ROI spanning the metastasis. The used approach enabled comparing features from the entire tumor, which averages out differences, with features from smaller tumor regions to better capture tumor heterogeneity and classify each metastasis individually. 92 patients with newly diagnosed stage IV CRC treated with a standard first-line chemotherapy were retrospectively evaluated. The patients had at least one measurable secondary liver lesion, as defined by the RECIST criteria (diameter >10 mm). For each segmented metastasis, the radiologist measured the longest diameter at baseline and again after 3 months of therapy (time point 1). Based on changes in size, the metastases were classified as non-responders (R−) or responders (R+). Five training sets were created from dataset 1 (using the ROI approach), and two were extracted from dataset 2 (using the 3D approach). Three predictive models were selected for classification: Gaussian Naive Bayes (NB), Multilayer Perceptron, and SVM. For the ROI approach, SVM was excluded from the study due to its high computational demands and the large volumes of data that needed to be processed. The best-performing model was based on the 3D approach, which used a genetic algorithm (GA) for feature selection and an SVM algorithm for classification. The model’s accuracy was 61% on the training set, 72% on the test set, and 82% on the validation set. However, the ROI approach achieved 63% accuracy on the construction set and only 48% on the validation cohort. Based on these findings, the authors suggest that the 7 × 7 ROIs do not adequately capture the heterogeneity of metastases, likely because they are too small to capture such tumor characteristics (Defeudis et al., 2021). However, several factors, such as segmentation modality, CT instrumentation and protocols, and image reconstruction algorithms, have been shown to affect the reproducibility and reliability of radiomics features (Engstrom et al., 2003; Andre et al., 2015). To overcome these limitations, Giannini et al. hypothesized that delta-radiomics, which analyzes changes in radiomic features extracted from CT scans across multiple time points, may help address limitations of conventional radiomics models (Yothers et al., 2011). This is crucial since it would help predict how CRC patients with liver metastasis (LMCRC), accounting for approximately 20% of CRC (Colucci et al., 2005) and who typically face a poor prognosis, with a median OS of less than 20 months (Soveri et al., 2019), respond to first-line FOLFOX chemotherapy. Therefore, Giannini et al. sought to predict the response of LMCRC patients to first-line FOLFOX chemotherapy using a delta-radiomics score, derived by subtracting baseline CT radiomic features from those obtained at time point one (TP1). The retrospective study collected data from real-world patients referred to two comprehensive cancer centers located in northern Italy, and the delta-radiomics predictions were compared against RECIST 1.1 results (Kerckhove et al., 2017). Using the median progression-free survival data from the most relevant phase III clinical trials on first-line chemotherapy for metastatic CRC (de Gramont et al., 2000; Douillard et al., 2014; Bokemeyer et al., 2009), the lesions were divided into two groups: non-responders (R-) and good responders (R+). Among all ML methods tested, the DT model achieved the highest performance, reaching 86% accuracy on the validation set, outperforming the other methods, which ranged from 63% to 77%. The delta radiomics signature accurately predicted long-term responses in LMCRC patients, achieving 97% accuracy in the training dataset and 86% in the validation dataset. Importantly, the signature correctly predicted R-lmCRC that were mistakenly classified as R+ by the RECIST method at TP1. In fact, it confirmed 106 out of 114 lesions as R-with 93% specificity, compared to only 76 lesions with 67% specificity using RECIST. The findings of this study are of key clinical importance for identifying patients predicted to have a short-term overall response, particularly by pinpointing unresponsive lesions in patients with a heterogeneous response. Liver lesions predicted as resistant at the first follow-up in the context of a mixed response could be selected for biopsy and further pathological reassessment. This may potentially uncover distinct genetic traits prompting the use of alternative targeted therapies or ablative interventional radiology procedures (Yothers et al., 2011).

As radiomics advances, combining PET/CT with other imaging techniques (like MRI) and incorporating multi-omics data (including genomic profiles) is becoming increasingly common. This data fusion enhances visualization strategies, providing additional biological insights and enabling a more comprehensive understanding of tumor behavior (Li et al., 2024). Advanced ML and DL algorithms are subsequently applied to interpret these features, facilitating a more efficient analysis of extensive imaging datasets (Gillies et al., 2016). For instance, Wei et al. conducted a retrospective study investigating the effectiveness of a DL-based radiomics model for differentiating responders from non-responders to first-line chemotherapy in colorectal liver metastases, using contrast-enhanced multidetector computed tomography (MDCT). Chemotherapy regimens included oxaliplatin-based treatments (CAPEOX or mFOLFOX6) or irinotecan-based treatments (FOLFIRI or XELIRI). Tumor response evaluation was performed within 8–12 weeks after initiation of the first cycle of chemotherapy using MDCT. Four main models were developed to predict chemotherapy response in CRLM: a clinical model, a qualitative model, a DL-based radiomics model, and a combined model. The carcinoembryonic antigen (CEA) level, a biomarker associated with advanced disease and reduced long-term survival in patients with LMCRC, was the only predictive factor that displayed significant differences between responders and non-responders (Wei et al., 2021; Kim et al., 2019). Interestingly, the combined DL radiomics model, which integrated the CEA level with DL-based fusion radiomics signatures, achieved the highest AUC of 0.935 in the training cohort and 0.830 in the validation cohort. In addition, stratification analysis revealed that the combined DL radiomics model demonstrated consistent performance in response prediction across subgroups defined by tumor number, tumor size, CEA level, and differentiation grade. Thus, DL-based radiomics models derived from MDCT were effective at predicting chemotherapy response in CRLM, outperforming conventional clinical factors and texture analysis (Wei et al., 2021). Interestingly, other biomarkers associated with CRC resistance have been evaluated in various algorithms. For example, it is well known that carcinoma-associated fibroblasts (CAFs) play a pivotal role in the TME, promoting resistance to cancer therapies. In addition, CAFs can trigger the nuclear translocation of AKT, survivin, and ERK, promote the phosphorylation of AKT and ERK, and upregulate survivin expression (Goncalves-Ribeiro et al., 2016). Growing evidence also suggests that chemotherapy resistance may be linked to the activation of two crucial Ras downstream pathways: the MAPK and the PI3K-AKT-mTOR signaling pathways (McCubrey et al., 2007; Pritchard and Hayward, 2013; West et al., 2002). Also, survivin is hypothesized to be a downstream effector of the MAPK (Oun et al., 2018; Tavares et al., 2020), or PI3K-AKT-mTOR signaling pathways (Gu et al., 2019), and its overexpression has been linked to drug resistance across multiple cancer types (Zaffaroni and Daidone, 2002; Nam et al., 2017; Cheung et al., 2013). Based on this, Gu et al. developed a predictive model of α-SMA, p-AKT, p-ERK, and survivin expression in CRC patients to identify those who may have intrinsic resistance to the oxaliplatin plus 5-FU chemotherapy regimen. Expression levels were assessed by immunohistochemistry of paraffin-embedded patient samples (Gu et al., 2019). Consequently, a predictive model was developed using a probabilistic neural network (PNN), which combines the strengths of radial basis networks and classical probability density estimation, offering significant benefits for pattern classification (Stubb et al., 2009). The study demonstrated that in stage IV CRC patients, high α-SMA expression was associated with significantly shorter progression-free survival (PFS) (5.5 vs. 15.0 months); however, its impact on OS was not statistically significant (30.7 vs. 35.0 months). However, high survivin expression was strongly associated with both reduced PFS (5.5 vs. 15.0 months) and OS (15.0 vs. 50.0 months). No significant correlations were found between p-AKT or p-ERK expression on either PFS or OS. In stage III CRC patients, there was no association between the expression of α-SMA, p-AKT, survivin, or p-ERK and PFS, as PFS times varied minimally and were not statistically significant. Similarly, there was no significant link between the expression of p-AKT, survivin, or p-ERK and OS. The predictive model based on the overexpression of α-SMA, p-AKT, p-ERK, and survivin demonstrated high efficacy (81.3%) and accuracy (81.8%). This suggests that patients with high expression of these parameters may be intrinsically resistant to an oxaliplatin plus 5-FU regimen (Gu et al., 2019). Table 1 summarizes the different methods used to predict chemotherapy response in CRC, highlighting the type of chemotherapy, AI algorithm/model, input features, training set metrics, test set metrics, and overall predictive performance.

**TABLE 1:** Summary of artificial intelligence (AI) models for predicting chemotherapy response in CRC.

Chemotherapy	AI algorithm/Model	Input features	Training set metrics	Test set metrics	Overall predictive performance	Year	Ref
Molecular biomarkers learning models							
First-line FOLFOX or FOLFIRI	RF and SVM classifiers trained with features selected by LASSO and VarSelRF.	Microarray differentially expressed (DE) gene profiles	RF best (across FOLFOX, mFOLFOX, mFOLFIRI) ACC 1.00 SE 1.00 SP 1.00	RF best FOLFOX: ACC 1.00, SE 1.00, SP 1.00, AUC 1.00 mFOLFOX: ACC 0.93, SE 1.00, SP 0.87, AUC 0.92 mFOLFIRI: ACC 0.96, SE 0.92, SP 1.00, AUC 0.94	Random Forest consistently achieved the best results across validation and independent test cohorts, showing near-perfect predictive performance across all three regimens	2023	Amniouel and Jafri (2023)
First-line FOLFOX chemotherapy	Top 3 machine learning algorithms were RF, SVM, and Neural Network Algorithms	Microarray differentially expressed (DE) gene profiles	During Cross-Validation, SVM mean values: ACC 0.7 SE 0.55 SP 0.75 YI 0.3	SVM best SE 0.90 (95% CI 0.67–0.98) SP 0.69 (95% CI 0.39–0.90) AUC 0.827 (95% CI: 0.670–0.984, P < 0.01)	Response vs. non-response: AUC = 0.877, 95% C.I. 0.747–1.00, 85% SE, 69.2% SP Improved survival: HROS = 0.358, 95% C.I. 0.178–0.717 in upregulation of MLK1 Improved survival: HROS = 0.563, 95% C.I. 0.336–0.943 in upregulation of CCDC124	2020	Lu et al. (2020)
First-line FULV or FOLFOX chemotherapy	Colon Oxaliplatin Signature (COLOXIS) machine learning (ML) model	Metagenes representing tumors	_	_	COLOXIS + associated with poorer outcomes on FULV (HR 1.52, 95% CI 1.07–2.15; P = 0.017) COLOXIS + derived benefit from oxaliplatin (HR 0.65, 95% CI 0.48–0.89; P = 0.0065; int P = 0.03) COLOXIS– showed no benefit (HR 1.08, 95% CI 0.77–1.52; P = 0.65)	2024	Chen et al. (2024a)
Oxaliplatin-based chemotherapy	LASSO, DT, RF, and SVM	Long noncoding RNAs (lncRNAs)	_	​	Models predictive of oxaliplatin sensitivity with high accuracy; AUC range for the four ML models was 0.794–0.880	2023	Zhou et al. (2023)
Oxaliplatin-based chemotherapy combined with bevacizumab	FOLFOXai	Next-generation sequencing (NGS) data	_	_	FOLFOXai predictive of OS FOLFOX HR 0.63 (P = 0.04) FOLFOXIRI HR 0.48 (P = 0.02)	2021	Abraham et al. (2021b)
Cisplatin, carboplatin, and oxaliplatin	SVM, GI50 threshold-dependent and threshold-independent machine learning models	Biochemically-inspired gene signatures	_	_	Oxaliplatin: Ensemble ML (1-year prediction) Mean error −0.12 ± 2.7 HU Accuracy: 60% disease-free (N = 88) 73% recurrent (N = 11) (After 1 year)	2019	Mucaki et al. (2019)
First-line FOLFOX chemotherapy	Iterative supervised learning method IML	Gene expression data	SE 97.6% SP 100%	_	Stage IV CRC HR 2.6 (P = 0.02) 3-year survival: Responders 61.9% (95% CI 41.9–91.4) Non-responders 18.8% (95% CI 6.8–52.0) Stage III CRC Predicted non-responder vs. responder HR 2.36 (P = 0.02)	2021	Tian et al. (2021)
First-line oxaliplatin or irinotecan-based regimens	Cytokine-based prognostic classifier CBPC: SVM + Cox PH	Cytokine expression data	OS prediction Accuracy 0.810 Sensitivity 0.835, Specificity 0.800 Median OS: high-risk 18.4 months, low-risk 51.7 months	OS prediction ACC 0.749 SE 0.831, SP 0.667 Median OS: high-risk 16.8 months, low-risk 55.9 months	Classifier maintained significant prognostic value in the combined cohort (n = 134), stratifying patients into high-risk vs. low-risk OS groups (P < 0.001)	2015	Chen et al. (2015)
First-line FOLFOX	RF	Gene expression data	SE 81.4% SP 85.1% With RF outlier removal: SE 91.3% SP 95.6% out-of-bag classification accuracy: 80.2%	SE 80.0% SP 92.8% ACC 69.2%	In the independent test cohort: RF classifier significantly stratified survival outcomes, with 2-year OS of 69.8% for predicted responders (N = 13) compared with 38.6% for non-responders (N = 16) (*P* = 0.0329)	2012	Tsuji et al. (2012)
Imaging biomarkers learning models							
First-line FOLFOX	DT	Delta radiomics signature: Contrast-enhanced computed tomography (CT) scans at differential time points	Per-lesion approach ACC: 97% SE 99% SP 94% PPV 95% NPV 99%	Per-lesion approach ACC: 86% SE 85% SP 92% PPV 90% NPV 87%	Delta-radiomics decision tree predictive of oxaliplatin response; AUC 0.99 (95% CI 0.97–1.0, training), AUC 0.93 (95% CI 0.87–0.96, validation)	2022	Giannini et al. (2022)
Oxaliplatin plus 5-fluorouracil (5-FU)-based regimens	Probabilistic Neural Networks PNNs	Immunohistochemical evaluation evaluation: α-SMA p-AKT p-ERK Survivin	ACC 83.7% (Best, 4 inputs)	ACC 92.9% (Best, 4 inputs)	PNN identified responders vs. non-responders using 4 protein markers; high α-SMA and survivin expression associated with poor progression-free and overall survival Prediction ACC 85.7% (Best, 4 inputs)	2019	Gu et al. (2019)
Oxaliplatin (CAPEOX or mFOLFOX6) or irinotecan (FOLFIRI or XELIRI) based regimens	ResNet10-based DL radiomics	Contrast-enhanced multidetector computed tomography (MDCT) images	DL-based model AUC 0.903 (95% CI 0.851–0.955) DL-based model + CEA AUC 0.935 (95% CI 0.897–0.973)	DL-based model AUC 0.820 (95% CI 0.681–0.959) DL-based model + CEA AUC 0.830 (95% CI 0.688–0.973)	DL-based radiomics significantly outperformed LASSO (AUC 0.745/0.598) and CEA alone (AUC 0.489/0.558); robust predictor of chemotherapy response in CRLM CEA provides a slight boost to DL-based model	2021	Wei et al. (2021)
First-line FOLFOX or FOLFIRI	Best results were obtained in the per-lesion analysis from the 3D approach using a SVM as classifier	CT scans at baseline 75 radiomics features	Per-lesion 3D set SE: 76%, SP: 67%, ACC: 72%, 75% NPV, PPV: 69% Per-patient 3D set SE: 80%, SP: 68%, NPV: 78%, PPV: 69%	Per-lesion approach SE: 61%, SP: 60%, NPV: 64%, PPV: 57% Per-patient approach: SE: 41%, SP: 21%, NPV: 23%, PPV: 38%	Independent validation confirmed only moderate predictive performance. 3D per-lesion SVM performed best (ACC ∼61%), while per-patient validation was poor	2021	Defeudis et al. (2021)

ACC, accuracy; AUC, area under the receiver operating characteristic curve; CI, confidence interval; DL, deep learning; DT, decision tree; HR, hazard ratio; HROS, hazard ratio for overall survival; HU, hounsfield unit; IML, integrative machine learning; LASSO, least absolute shrinkage and selection operator; MDCT, multidetector computed tomography; ML, machine learning; NPV, negative predictive value; OS, overall survival; PNN, probabilistic neural network; PPV, positive predictive value; RF, random forest; SE, sensitivity; SP, specificity; SVM, support vector machine; YI, youden index.

### 2.6 Commonly reported biomarkers across studies

Cross-comparison of signatures across studies is illustrated in Supplemental data, Table 1. The APPB2 gene was the only gene consistently reported by more than one study, suggesting it may serve as a shared biomarker of oxaliplatin response. Lu et al. reported APPBP2 to be downregulated in responders to oxaliplatin therapy, and Tsuji et al. reported APPBP2 to be a predictor of poor response to oxaliplatin therapy. However, the limited overlap between other signatures likely reflects heterogeneity in study design and patient cohorts. The absence of uniform clinical endpoints, such as progression-free survival, overall survival, or pathological response, further complicates cross-study interpretation. Additionally, differences in bioinformatic preprocessing pipelines contribute to variability in the reported biomarkers. This limited overlap underscores the current lack of standardization in AI-driven biomarker discovery and highlights the need to establish standardized protocols and implement cross-institutional data sharing. Such actions could enhance reproducibility and facilitate the development of clinically actionable biomarkers to guide oxaliplatin-chemotherapy-based treatments.

## 3 Oxaliplatin-induced toxicity in patients with CRC - the role of AI

Oxaliplatin-based chemotherapy (e.g., FOLFOX) is associated with several side effects, including peripheral neuropathy, gastrointestinal (GI) toxicity, and hematological toxicity (Oun et al., 2018; Mauri et al., 2020). Oxaliplatin-induced peripheral neuropathy (OIPN) is a common and serious side effect that reduces patients’ quality of life and can impact long-term survival (Tavares et al., 2020). Symptoms often begin as tingling or numbness in a “glove and stocking” distribution (Desforges et al., 2022; Miltenburg and Boogerd, 2014; Stubb et al., 2009; Hershman et al., 2014). Approximately 90% of patients experience acute neuropathy following oxaliplatin therapy, while chronic neuropathy affects 13%–70% patients (Hershman et al., 2014; Pachman et al., 2015; Kelley and Fehrenbacher, 2017; Song et al., 2017). Luckily, acute neuropathy is generally reversible and transient, often resolving within days to weeks, and rarely requires treatment cessation. Severe chronic OIPN, on the other hand, is cumulative and frequently irreversible, and may necessitate dose reduction or discontinuation of treatment, thus limiting therapeutic efficacy (Matsuda et al., 2023; Yang et al., 2021; Teng et al., 2022). Additionally, oxaliplatin is associated with several gastrointestinal (GI) adverse effects, including nausea, vomiting, diarrhea, mucositis, and stomatitis (Oun et al., 2018). Among these, nausea and vomiting are the most common and typically occur within 24 h to 5 days following infusion (Fleishman et al., 2012). Unfortunately, stomatitis can be painful and debilitating, affecting a patient’s ability to eat and speak (Oun et al., 2018; Fleishman et al., 2012). GI toxicities (nausea, vomiting, diarrhea, etc.) are typically reversible upon cessation of therapy and can be actively managed with supportive care (antiemetics, antidiarrheals) (Oun et al., 2018). Severe, uncontrolled symptoms may temporarily require delaying or reducing the subsequent dose, but rarely permanent cessation (Teng et al., 2022; Lee et al., 2024). Additionally, hematological side effects associated with oxaliplatin use include myelosuppression, leading to leukopenia, neutropenia, thrombocytopenia, and anemia (Oun et al., 2018). Hematological toxicities are usually reversible but can be life-threatening (e.g., febrile neutropenia). Severe myelosuppression often mandates a dose reduction or treatment delay until blood counts recover, but typically not complete cessation of treatment (Hershman et al., 2014).

Given the significant side effects associated with oxaliplatin use, the section below highlights the current knowledge of the mechanisms underlying oxaliplatin-induced toxicities, available prophylactic strategies, and the use of ML models to predict toxicities in affected patients. However, AI use extends far beyond predicting adverse outcomes. AI tools, including advanced ML models, are being actively deployed not only to predict toxicities in affected patients but also to design and identify new therapeutic strategies. Specifically, AI can be used to screen large chemical libraries and model molecular interactions to pinpoint novel transporter inhibitors or protective agents that can effectively mitigate neurotoxicity, positioning AI as a powerful tool for *de novo* drug design and personalized supportive care.

### 3.1 Oxaliplatin-induced peripheral neuropathy

#### 3.1.1 Molecular and cellular mechanisms

Oxaliplatin enters highly sensitive neurons, particularly in dorsal root ganglia (DRG), via the hOCT2 transporter (Yamane et al., 2025; Wakai et al., 2022), which has been shown to play a key role in OIPN pathogenesis (Yang et al., 2021; Carozzi et al., 2015). Once inside, oxaliplatin damages neurons by forming DNA adducts, which disrupt cellular function (Matsuda et al., 2023). Cold-sensitivity, a hallmark OIPN symptom, is caused by overactivation of the TRPM3 receptor (Descoeur et al., 2011; Deuis et al., 2013; Poupon et al., 2018; Sittl et al., 2012). Therefore, OIPN results from a cascade of events starting with hOCT2 entry, followed by DNA damage and receptor overactivation (Yang et al., 2021). Interestingly, AI-assisted studies suggest that oxaliplatin primarily targets the cell body of neurons rather than their axons (Matsuda et al., 2023). Here, AI combines vast datasets from imaging, electrophysiology, and molecular profiling. Thanks to ML models, scientists have uncovered predictive toxicity patterns that often slip through the cracks of traditional analyses (Matsuda et al., 2023). These models have consistently associated early pathological signs, such as mitochondrial swelling, ribosomal stress, and disrupted transcriptional networks with the DRG soma rather than the distal axons (Matsuda et al., 2023; Poupon et al., 2018). Moreover, DL algorithms analyzing neuronal morphology have shown that structural degeneration in the soma occurs before measurable changes in the axons, indicating that the cell body is the first area to sustain injury (Matsuda et al., 2023). Through feature-importance ranking, AI systems have identified somatic pathways involved in RNA processing, protein synthesis, and metabolic stress as crucial factors in OIPN vulnerability, thereby shifting the focus from the conventional “dying-back” axonopathy to a soma-initiated neurotoxicity perspective (Argyriou, 2015).

### 3.2 Prophylactic strategies and mechanisms

One strategy to prevent OIPN is blocking the hOCT2 transporter (Yamane et al., 2025). Existing drugs such as cimetidine and proton pump inhibitors (PPI) have shown potential to reduce neuropathy in mice by inhibiting hOCT2 (Ciarimboli et al., 2010; Hiramatsu et al., 2020; Kobayashi et al., 2023; Wakai et al., 2022). While clinical validation for OIPN prevention of cimetidine remains lacking, with evidence currently limited to preclinical studies, several retrospective clinical studies provide supportive evidence for the protective effects of PPI in patient populations. For instance, a recent retrospective cohort study of 1,015 patients receiving oxaliplatin-based chemotherapy shows that concomitant PPIs use associate with a significant reduction in oxaliplatin discontinuation owing to OIPN (adjusted hazard ratio = 0.568, 95% CI 0.344–0.937, p = 0.0269), with propensity score-matched analysis confirming this protective effect (adjusted hazard ratio = 0.478, 95% CI 0.273–0.836, p = 0.0096) (Mine et al., 2025). Similarly, in another retrospective analysis of 217 CRC patients, concomitant PPI use significantly reduced the incidence of grade ≥2 CIPN (odds ratio = 0.054, p < 0.001) and prolonged time to onset of neuropathy without affecting therapeutic efficacy (Kobayashi et al., 2023). Additionally, an analysis of the FDA Adverse Event Reporting System (FAERS) database revealed that the reporting rate of peripheral neuropathy was lower among oxaliplatin-treated patients concurrently using PPI than among those not using them (3.06% versus 6.48%, p < 0.001, reporting odds ratio 0.44) (Mine et al., 2022).

Other drugs show promise through different protective pathways, though the conflicting existing data about their potential benefits, such as for statins, remain mixed and a significant challenge. A multicenter retrospective observational study of 2,657 patients found that in propensity score-matched CRC patients, the incidence of grade ≥2 OIPN was significantly lower among statin users compared to non-users (19.8% versus 28.3%, p = 0.029), with a reduced rate of oxaliplatin dose reduction observed in the statin group (Takechi et al., 2025). However, in another single-center observational study of 224 CRC patients receiving adjuvant CAPOX therapy, no significant difference in OIPN incidence was observed between statin and non-statin users (94% versus 95%, p = 0.67), suggesting that the protective effect of statins may be context-dependent (Okamoto et al., 2023). Interestingly, in mice, statins exert neuroprotective properties by activating the glutathione S-transferase (GST) system, which helps combat oxidative stress (Aizawa et al., 2024). AI-assisted pathway analysis showed that the GST system is also closely linked to inflammatory regulation by identifying co-expressed inflammatory markers and GST-related detoxification genes as strongly correlated across transcriptomic datasets (Aizawa et al., 2024), indicating that GST activation may reduce both oxidative and inflammatory components of OIPN. Importantly, statins do not interfere with oxaliplatin’s anticancer mechanisms.

Interestingly, researchers developed the first ML-driven Quantitative Structure-Activity Relationship (QSAR) model specifically to predict hOCT2 blockers (Yamane et al., 2025; Peng et al., 2021; Lo et al., 2018). The model used an RF classifier trained on curated datasets of known hOCT2 inhibitors and non-inhibitors, and incorporated molecular descriptors such as extended-connectivity fingerprints (ECFP), physicochemical parameters (e.g., logP, molecular weight), and topological indices (Yamane et al., 2025). The model performed well, with an accuracy of 0.87 and an F1 score of 0.89, confirming its potential for drug discovery capabilities (Yamane et al., 2025). Using the QSAR model, the researchers screened over 11,000 compounds and identified 162 FDA-approved drugs as potential hOCT2 inhibitors (Yamane et al., 2025). Dequalinium, an antimicrobial agent, was validated as a potent hOCT2 inhibitor with an IC_50_ of 11.37 ± 5.32 nM, effectively blocking hOCT2-mediated oxaliplatin transport, suggesting it could be a prophylactic candidate to prevent OIPN (Yamane et al., 2025; Lo et al., 2018). Yet, clinical validation of dequalinium-based OIPN prevention remains lacking, with evidence currently limited to preclinical studies. Collectively, these findings suggest multiple ways to prevent OIPN, either by blocking drug entry or strengthening cellular defenses, and combining these strategies may provide greater protection. Given that most findings are based on preclinical data, future clinical studies are warranted to validate the efficacy and safety of these pharmacological interventions for preventing OIPN in patients receiving oxaliplatin-based chemotherapy.

Table 2 summarizes key mechanisms involved in OIPN and their proposed pharmacological interventions.

**TABLE 2:** Key mechanisms of OIPN and their proposed pharmacological interventions.

Mechanism of OIPN	Proposed intervention	Mechanism of action	Observed effect/Concentration	Ref.
hOCT2-mediated uptake of Oxaliplatin into DRG neurons	Dequalinium	Potent hOCT2 inhibitor	Inhibited hOCT2-mediated oxaliplatin transport (IC50 = 11.37 ± 5.32 nM)	Yamane et al. (2025)
	Cimetidine	Typical hOCT2 inhibitor	Reduced OIPN in mice when administered intravenously at 30 mg/kg prior to oxaliplatin (5 mg/kg)	Sprowl and Sparreboom (2014)
	Proton Pump Inhibitors	Off-target hOCT2 inhibition	Ameliorated OIPN in mice at clinically relevant doses. Omeprazole 20 mg/kg (i.p.) significantly prevented mechanical hypersensitivity	(Yamane et al., 2025)
Direct cellular damage (DNA adducts, apoptosis) and inflammatory response	Statins (Simvastatin, Atorvastatin, Rosuvastatin)	GST pathway activation (redox modulation, anti-inflammatory)	Mitigated mechanical allodynia in mice (10 mg/kg), without affecting anti-tumor activity	Aizawa et al. (2024)
TRPM3 activation leading to cold allodynia	(No direct intervention identified)	(Not applicable)	Oxaliplatin induces TRPM3 activation	(Aloi et al., 2022)
Soma reduction and contour deformation	(Not directly addressed by intervention)	(Not applicable)	Observed in DRG neurons treated with oxaliplatin (10 μM, 100 µM)	Yamane et al. (2025)
Axonal aggregation	(Not directly addressed by intervention)	(Not applicable)	Observed in axons treated with oxaliplatin (low/high concentrations)	(Sprowl and Sparreboom, 2014)

OIPN, Oxaliplatin-Induced Peripheral Neuropathy; hOCT2, human Organic Cation Transporter 2; DRG, dorsal root ganglia; IC50, Half Maximal Inhibitory Concentration; GST, Glutathione S-Transferase; TRPM, Transient Receptor Potential Melastatin 3; i.p., intraperitoneal.

### 3.3 Association of body composition with oxaliplatin pharmacokinetics and toxicity in patients with colorectal cancer

Dosing of chemotherapy agents is based on body surface area (BSA); however, BSA is a poor indicator of drug metabolism and clearance. In a prospective, multicenter observational study, Li et al. demonstrated that plasma levels of 5-FU were subtherapeutic in 60.6% of patients, supra-therapeutic in 19.1%, and within the desired therapeutic range in only 20.3% (Li et al., 2023). Thus, BSA fails to achieve optimal drug exposure and does not capture the pharmacokinetic variability existing among individuals. Growing evidence suggests that assessing body composition could help refine chemotherapy dosing, reducing toxicity while maintaining treatment effectiveness (da Silva Dias et al., 2021). Oxaliplatin, a complex platinum-based drug with both lipophilic and hydrophilic properties, distributes predominantly into adipose tissue. Conversely, fluoropyrimidines such as 5-FU are hydrophilic and are taken up more readily into SM (Nam et al., 2017; Cheung et al., 2013). These differences further highlight how variations in body composition can significantly influence drug distribution, even among patients with the same BSA, and may therefore provide valuable insights for optimizing dosing of both agents (Chen et al., 2015).

For example, patients with low lean body mass (LBM) have a lower drug distribution volume (V_d_), leading to higher blood concentrations. In contrast, those with high total adipose tissue (TAT) tend to have elevated peak concentrations (C_max_) (Williams et al., 2021). The low LBM/high TAT phenotype was strongly associated with a 45% increased risk of experiencing grade III-V GI and hematologic chemotoxicity and accounted for 11%–17% of oxaliplatin pharmacokinetic variability (Williams et al., 2021). While this data further confirms that conventional BSA-based dosing is a poor predictor, it reveals the urgent need for personalized dosing based on body composition to improve patient safety and outcomes during oxaliplatin chemotherapy. Similarly, a recent study found that patients with a low skeletal muscle index (SMI) (sarcopenia) had a higher incidence of severe hematologic toxicity (39% vs. 24%) during FOLFOX therapy, especially neutropenia (31% vs. 15%) (Lei et al., 2025). This was particularly pronounced in males with low SMI (46% vs. 21%) (Lei et al., 2025), indicating that sex-specific differences and body composition influence oxaliplatin toxicity (Supplemental data, Table 2). Interestingly, low SMI was also associated with reduced 5-FU dose intensity, potentially leading to suboptimal treatment (Lei et al., 2025). This suggests that patients with lower muscle mass may receive suboptimal treatment due to unnecessary dose reductions. The data clearly demonstrate a significant association between low SMI and an increased incidence of severe-grade neutropenia (grade 3 or higher), especially in male patients. This indicates that sarcopenia is not just a comorbidity but a direct risk factor for severe hematologic side effects and compromised treatment delivery. Low SMI can lead to altered drug pharmacokinetics and metabolism, which then results in higher hematologic toxicity and subsequent dose reductions. The findings suggest that baseline skeletal muscle assessments, readily obtainable from standard-of-care CT scans, could provide crucial information to guide dose modifications and thereby prevent severe grade neutropenia (grade III or higher) (Lei et al., 2025).

Based on that notion, Besson et al. used AI-mediated 3D body composition to evaluate the impact of SM and adipose tissue on chemotherapy-related adverse effects, thereby developing body composition-based dosing guidelines for chemotherapy agents. To achieve that, a retrospective study of 242 patients with rectal cancer was carried out with 33 patients receiving total neoadjuvant chemotherapy (either four cycles of CAPOX or six cycles of FOLFOX) and 209 patients being treated with long-course chemoradiotherapy (Chen et al., 2015). Tissue volume (cm^3^) and average radiodensity (HU) for SM, IMAT, Visceral adipose Tissue (VAT), and subcutaneous adipose tissue (SAT) were measured using a pre-trained, validated in-house AI segmentation model (Gu et al., 2019). The authors investigated the relationship between SM and adipose tissue to develop a novel scoring index, calculated as SM/(VAT + IMAT). Results show that in rectal cancer patients receiving standard neoadjuvant therapy, chemotherapy toxicity was more frequent in female patients. Dose reduction or early cessation was required in 26.9% of female patients, compared with 15.9% of men. Body composition varied between genders: males had greater SM and VAT volumes, whereas females had greater SAT volumes. Such differences in body composition contribute to the well-established gender-based variation in chemotherapy toxicity (Mauri et al., 2020). In addition, females with increased VAT density and reduced VAT volume had a higher incidence of dose modification. Therefore, VAT volume was proposed as a marker of systemic malnutrition, with low volumes indicating body composition changes associated with cancer cachexia. Female patients with low VAT volumes were also found to be more likely to have concurrent sarcopenia (42% vs. 19%, p = 0.038) than females with greater VAT volume. The two strongest predictors were VAT density and the SM: VAT + IMAT volume ratio, with an AUC value of 0.699 and 0.651, respectively. A cut point of 1.61 for the SM: VAT + IMAT volume ratio was identified, yielding a sensitivity of 76% and specificity of 61%. Values above this cut point had a 3.2-fold higher relative risk (95% CI 1.3–7.9, p = 0.01) of requiring dose modification (Chen et al., 2015).

Using AI to analyze the entire lumbar spine (L1–L5) improved prediction of chemotherapy dose-limiting toxicity in CRC patients (AUC 0.75 vs. 0.65 for single-slice analysis) (Chen et al., 2015). Of clinical relevance, peripheral neuropathy was the most common toxicity (43.8%) in this group (Chen et al., 2015). Together, these findings reinforce that muscle wasting is a critical predictor of poor outcomes during chemotherapy (Li et al., 2012). AI-derived body composition metrics could therefore become essential biomarkers for guiding treatment and prognosis.

### 3.4 AI in toxicity surveillance

Traditional toxicity monitoring, which relies on laboratory tests and examinations, can miss early signs of oxaliplatin side effects. AI offers a proactive solution by enabling continuous, real-time monitoring and early detection, which are essential for detecting oxaliplatin-related toxicity and can prevent long-term damage, ultimately improving outcomes (Yang et al., 2021; Jiang et al., 2025). Fortunately, AI applications have shown great potential across various areas, including predicting chemotherapy toxicities. For instance, in peripheral neuropathy, AI-powered microphysiological systems (MPS) can identify subtle neuronal changes, such as cell body shrinkage, before symptoms are noticeable. It has been demonstrated that DT models analyze high-resolution images of neurons to flag future risk of neuropathy (Matsuda et al., 2023). Matsuda et al. utilized an MPS integrated with DL-based soma and axonal AI models to evaluate the *in vitro* toxicity and mechanism of action (MoA) of oxaliplatin. The study demonstrated that oxaliplatin induced significant soma toxicity at 10 µM (32.5% ± 7.2%) and 100 µM (39.3% ± 10.8%), both of which were significantly higher than the DMSO control. In contrast, axonal toxicity levels were 28.5% ± 6.8% at 10 μM and 24.2% ± 5.6% at 100 μM, values not significantly different from DMSO, though slightly above the positive threshold line (Matsuda et al., 2023). The DL-based graphical MoA classification revealed a predominant shift along the y-axis, indicating soma-specific damage rather than axonopathy. These findings highlight the potential of AI-driven MPS systems for early neurotoxicity detection, MoA classification, and in vitro-to-in vivo extrapolation (IVIVE), offering a robust framework for mechanistic understanding and predictive toxicity assessment of chemotherapeutic agents such as oxaliplatin (Matsuda et al., 2023).

Furthermore, biomedical text mining is a fast-growing field that uses NLP to extract insights from vast volumes of literature (Rocca et al., 2023). It identifies key biological entities and their relationships, creating predictive models to understand disease mechanisms better and advance drug development (Raparthi et al., 2023). NLP-based AI tools are increasingly used to predict molecular pathways in CIPN. For instance, Cascade Eye analyzed the scientific literature to map a network of 310 CIPN-associated molecules and genes, identifying 605 predicted interactions (Aizawa et al., 2024). It highlighted the involvement of the GST gene family, flagging specific genes (GSTM1 and GSTT1) as causal and (GSTM3 and GSTP1) as interactive, and predicted intricate relationships between these genes and interleukin-1β (IL-1β) (Aizawa et al., 2024). This AI approach goes beyond summarizing existing knowledge to generate new hypotheses, such as the potential neuroprotective role of GST-IL-1β interactions in statin therapy, accelerating discovery by connecting disparate research findings (Aizawa et al., 2024). Importantly, NLP-based predictions have also elucidated oxaliplatin-specific mechanisms, including strong text-mined associations linking GSTM1-null and GSTP1 Ile105Val polymorphisms to heightened susceptibility to oxaliplatin-induced neurotoxicity (Peng et al., 2013), as well as recurring mechanistic themes involving ion channel dysregulation (NaV1.6, TRPA1, TRPV4) and oxidative stress pathways (Velasco et al., 2021). Moreover, tools such as PubTator, LitVar, and BioBERT identified key gene-variant associations, such as GSTP1, ERCC1, and ERCC2 polymorphisms, with oxaliplatin neurotoxicity (Inada et al., 2010). In contrast, platforms such as CoPub and STRING reveal robust links to ion channel hyperexcitability (TRPA1, TRPV1, NaV1.8), neuroinflammation (IL-1β, TNF-α, NF-κB), oxidative stress (Nrf2, SOD2), and axonal degeneration pathways (SARM1) (McGuinness et al., 2024). Additionally, semantic-mining tools such as DrugR+ and SemanticMedline have repeatedly flagged several potential neuroprotective agents, including duloxetine, minocycline, N-acetylcysteine, and statins, demonstrating how NLP accelerates hypothesis generation and therapeutic discovery for CIPN (Chen et al., 2024b). This technology is revolutionizing the way hypotheses are generated and biomedical knowledge is interpreted.

Interestingly, AI has also been used in combination with saliva tests to identify patients with impaired drug metabolism who are at risk of severe side effects (Janiga et al., 2025). Similarly, AI-enhanced analysis of electrocardiograms and cardiac imaging can detect early signs of chemotherapy-induced cardiotoxicity, enabling timely intervention and prevention of irreversible cardiac damage (Cheng et al., 2023).

Beyond prediction, AI-driven systems are used for real-time surveillance, where wearable devices and smart sensors continuously monitor patient vitals, and AI models detect early warning signs of toxicity (Abdul Rasool Hassan et al., 2025). A DL method was developed to analyze images of DRG neurons and assess drug neurotoxicity using an MPS that separates neuronal bodies from their axons, enabling high-resolution imaging and focused toxicity analysis (Matsuda et al., 2023). Two AI models were trained to detect cell death in the neuronal body and axonal damage, thereby accurately detecting oxaliplatin toxicity at both 10 μM and 100 µM (Matsuda et al., 2023). These AI models detected toxic changes earlier and at lower drug concentrations than traditional methods, demonstrating their high sensitivity to chemotoxicity (Matsuda et al., 2023), a capability valuable for early drug screening or clinical dose adjustments. By plotting predicted toxicity probabilities for the neuronal body against those for axons, drugs could be classified by their specific mechanisms of damage. For example, oxaliplatin primarily affected neuronal bodies, while paclitaxel damaged both axons and neuronal bodies, and vincristine mainly affected axons (Matsuda et al., 2023). This dual-model approach improves the precision of toxicity detection and provides deeper insights into how different drugs affect neurons. Moreover, AI-powered chatbots are emerging as tools for managing chronic diseases, including CRC, supporting self-care, providing health education, and offering mental health support through real-time, human-like interactions (Kurniawan et al., 2024; Altom et al., 2025). Users have reported high satisfaction and acceptance, highlighting the system’s user-friendliness (Kurniawan et al., 2024; Bin Sawad et al., 2022). Despite their promise, research is limited by the small number of high-quality studies, which often have short intervention periods and limited long-term follow-up (Kurniawan et al., 2024; Altom et al., 2025). Evidence on improving clinical outcomes is limited, and insufficient technical reporting makes reproducibility difficult (Kurniawan et al., 2024; Altom et al., 2025); hence, future studies must prioritize rigorous methods and standardized outcome measures (Kurniawan et al., 2024; Altom et al., 2025). Even with limitations, AI chatbots offer scalable, cost-effective support, particularly in resource-limited settings during CRC treatment. Their use must be guided by ethical considerations and data privacy (Kurniawan et al., 2024; Altom et al., 2025; Bin Sawad et al., 2022), and stronger evidence is needed for widespread clinical adoption in oncology therapy.

### 3.5 Translational potential: bridging AI and clinical practice

AI’s ability to integrate *in vitro*, *in vivo*, and *in silico* data alongside clinical variables, such as imaging biomarkers and patient-reported symptoms, yields a dynamic, multidimensional risk profile that goes beyond single-factor assessments and offers a unique framework for predicting toxicity (Froicu et al., 2024). For instance, several AI-enabled modalities provide complementary translational input: CT-based body composition analysis identifies sarcopenia and adiposity as indicators of drug tolerance; QSAR modeling screens for transporter-mediated neurotoxicity at the molecular level. Moreover, coupling Microphysiological systems (MPS) with DL enables in vitro-to-in vivo extrapolation and the detection of toxicity at clinically relevant concentrations (Aizawa et al., 2024). The use of NLP-driven data extraction from electronic health records further refines risk stratification by exposing undocumented symptom patterns. Correlating MPS-derived toxicity metrics with MRI-based tissue characterization, or integrating CT-derived body composition with pharmacokinetic models, offers a practical pathway to individualized dosing and may reduce reliance on animal models by anchoring predictions in human-relevant systems (Aizawa et al., 2024). The ultimate goal is not simply prediction but proactive personalization. Chatbot applications, though still nascent, may serve this latter function by enabling continuous symptom surveillance and facilitating timely clinical response. Yet, translation remains incomplete. Several substantial barriers, such as prospective validation in multicenter cohorts, interoperability with existing electronic medical records, and clinician acceptance, must be overcome before these approaches can be integrated into routine oncological practice.

AI will not replace clinical judgment, but it will increasingly inform and refine it, advancing a truly personalized, data-driven precision oncology.

## 4 AI in personalization of oxaliplatin-based chemotherapy dosing in CRC

In oncology therapeutics, particularly in the age of precision medicine, the objective is to administer the right drug(s) to the right patient at the right time (Westergren et al., 2025). However, a significant challenge remains in finding the correct dose for each patient. Typically, dosing is standardized based on data from a relatively small number of participants in early-phase clinical trials. In oncology, many clinical trials focus on combining approved drugs into new regimens to enhance efficacy. The maximum tolerated dose (MTD) and recommended phase II dose (RP2D) for these combinations are both determined from phase I trials (Kurzrock et al., 2021). Additionally, conventional oncology practice suggests that higher chemotherapy doses result in higher cancer cell kill and, therefore, potentially better treatment efficacy. Thus, the standard-of-care dose is guided by the MTD, leading to a high initial dose and subsequent dose reductions due to toxicity (Jimenez et al., 2022). This imposes a challenge since these standard doses fail to account for the wide variability in patient tolerance, which may be influenced by factors such as fragility, comorbidities, organ compromise, co-administered drugs, gender, age, race, and differences in drug metabolism (Peng et al., 2021; Burris et al., 2016; Zhou and Liu, 2000; Yeung et al., 2015; Dawson, 1940). As CRC management becomes increasingly complex, there is an urgent need for a revised, more accurate method for determining individual chemotherapy dosing. In fact, the 5-year survival rate for patients with advanced CRC has remained <8% due to the development of treatment resistance (Gravalos et al., 2007). The integration of AI and ML into personalized cancer treatment provides unprecedented opportunities for precision medicine. The strength of ML lies in its ability to integrate diverse data types, such as clinical and demographic factors, to build comprehensive patient profiles for therapy customization (Martinez-Garcia and Hernandez-Lemus, 2021). Also, ML has been applied to numerous pharmacometrics challenges, including pharmacokinetic and pharmacodynamic modeling and simulation, model-informed precision dosing, and systems pharmacology (Stankeviciute et al., 2023).

Generally, conventional combination therapy is designed by selecting drugs that simultaneously target multiple signaling pathways, and it is believed to achieve better treatment outcomes than monotherapy (Besson et al., 2025). However, drugs to be combined are frequently selected based on their effectiveness as single agents and are typically used at their maximum tolerated single-agent doses; thus, increasing the risk of toxicity and resistance (Pritchard et al., 2012). In addition, the synergistic effect of these drugs is dose-dependent. Therefore, administering drugs at incorrect dose ratios, even if the drugs themselves are appropriate, can lead to suboptimal responses (Weiss et al., 2015). To combat this, promising strategies for designing new drug combinations include pairwise drug predictions, systems biology-guided combination design, and *ex vivo* or disease modeling approaches, among others, all aimed at estimating how disease systems will respond to multiple drugs simultaneously (Zimmer et al., 2017; Fitzgerald et al., 2006; Katzir et al., 2019; Liu et al., 2018; Lam et al., 2018). However, optimizing both the drug and its dose simultaneously creates a search space that is too large for conventional high-throughput technology, which partly explains the high failure rates of drug trials and low response rates (Eggermont et al., 2018; Remus et al., 2023). Beyond the vast drug-dose search space, the complex mechanisms linking drug dosing, cellular omics, and physiological functions pose additional challenges for identifying the most potent regimens for patients (Wong et al., 2008; Zarrinpar et al., 2016). Employing AI offers solutions to these challenges as it is a mechanism-independent technology that can learn from data to optimize outcomes and account for disease heterogeneity, pharmacokinetics, comorbidities, and rapid changes to patient physiology (Kee et al., 2019). Therefore, Ding et al. investigated the inhibitory effects of a four-drug combination (AGCH: adriamycin, gemcitabine, cisplatin, and Herceptin) on the response of CRC tumors in a rat model (Ding et al., 2019). A few hundred experimental data points were used in an AI-based neural network to correlate drug-dose inputs with phenotypic outputs, such as tumor burden and toxicity markers (Al-Shyoukh et al., 2011). The phenotypic response surface (PRS) was unexpectedly smooth, indicating that the efficacies/toxicities have a simple relationship with the therapeutic inputs. Using a PRS-augmented AI platform, a novel four-drug combination (AGCH) was dynamically optimized in a rat tumor model. Rats were assigned to a control group (C1-C3, n = 3) receiving 0.1 mL PBS or to an individually optimized regimen (IOR) group (IOR1-IOR5, n = 5) receiving AGCH at ¼ MTD for each drug on days 1–7. PRS technology was then used to derive an IOR for each rat, which was administered on days 14–24. Despite genetic homogeneity of the rats and the cancer cell line, tumor responses to the uniform ¼ MTD AGCH regimen were highly variable, with only rat IOR4 showing a distinctly favorable response. Failing to determine optimized combination doses that account for genetics-related human heterogeneity and patient-specific physiological responses to therapy results in implicitly suboptimal treatment efficacy, which may explain the low response rates observed with ¼ MTD treatment. Remarkably, after implementing the IOR regimen, the AI-PRS-derived optimal doses for each rat led to a uniform treatment response, with all rats showing similar normalized tumor response rates. The average tumor response rate converged towards 0.00776/day (SD = 0.00291), closely matching the tumor response rate of IOR4, the best responder under ¼ MTD treatment. Interestingly, many drugs in the IOR regimen were administered at doses as low as 20% of the ¼ MTD, yet the regimen still achieved optimal efficacy. In addition, with response surfaces changing rapidly during treatment, PRS-based identification of these dynamic surfaces allows for continuous optimization of therapy throughout the course of treatment. AI-PRS optimization revealed that drug synergism and antagonism vary between subjects rather than being universal. For example, combined drug-drug interactions of IOR1, 2, 3, and 4 were synergetic; however, IOR5 was antagonistic (Ding et al., 2019). While the authors studied the ACGH regimen dosing on the AI-PRS platform, the platform is model-independent and can be applied to virtually all drug classes. This has been previously validated in multiple settings, including infectious diseases, clinical immunosuppression, and regenerative medicine (Weiss et al., 2015; Leber et al., 2017; Ehteshami Bejnordi et al., 2017; Chen et al., 2016; Harpaz et al., 2017; Cai et al., 2017; Haefeli et al., 2017). Thus, these findings support the applicability of this approach to optimizing oxaliplatin-based chemotherapy dosing in patients with CRC.

CURATE.AI is a personalized, AI-driven platform that builds a unique profile for each patient by linking intervention intensity (input) and a phenotypic result (output) for an individual, based exclusively on that individual’s data. By testing a range of input levels (such as drug doses) and linking them to measurable outcomes, the system calibrates itself to generate a personalized CURATE.AI profile. This profile then serves as a map, predicting responses to different inputs and recommending the intervention intensity that yields the best result. As the patient’s condition evolves (disease progression/recession, addition of new medications, dose changes, or other interventions), so does the CURATE.AI profile, which is dynamically recalibrated to ensure optimal care throughout the entire course of therapy (Blasiak et al., 2020a). CURATE.AI is not a conventional AI platform; instead, it builds on findings from a prior neural network study and reduces them to a simpler quadratic relation between treatment intensity (e.g., drug dosage) and phenotypic outcome (e.g., tumor markers) (Al-Shyoukh et al., 2011). This quadratic relationship reflects a non-linear pattern in which increasing intervention intensity may initially cause the response to rise, then fall, or to fall and then rise. This could be attributed to several factors, such as intra- and inter-individual differences in pharmacokinetics and pharmacodynamics (Kantae et al., 2017), genetic variations in cancer, tissue-drug interactions, and drug-drug interactions (Tan et al., 2011). CURATE.AI is undergoing clinical validation through prospective trials across several indications, including prostate cancer (NCT02711956) (Pantuck et al., 2018), cognitive rehabilitation therapy (NCT04848935) (Remus et al., 2023), and hypertension (NCT05376683) (Truong et al., 2024). Its applicability across diverse indications is enabled by simplifying the original neural network-based algorithmic process, incorporating derivatives of clinically relevant factors, and maintaining flexibility to adapt to different dose-response inputs (Zarrinpar et al., 2016; Kee et al., 2019; Blasiak et al., 2020b; Raczkowska et al., 2023; Truong et al., 2021). Blasiak et al. conducted a feasibility trial to evaluate the logistical and scientific practicality of CURATE.AI in providing dose recommendations for patients with advanced solid tumors. The trial included patients receiving single-agent capecitabine or capecitabine combinations with oxaliplatin (XELOX) or irinotecan (XELIRI). In the CURATE.AI version used in this study, the data inputs were: the total capecitabine dose administered during the cycle and the corresponding change in the response biomarker, expressed as a percentage of the pre-cycle level. A second-order polynomial regression model was employed to fit the available dose-response data pairs and generate a personalized profile. For subsequent treatment cycles, the dose-response data pairs were incorporated into the profile in the same manner, allowing the profile and CURATE.AI’s dose recommendations to evolve dynamically along the patient’s journey. In cases of systemic changes, such as a regimen change (e.g., from XELOX to XELIRI), CURATE.AI recommended “recalibration” dose(s) to obtain new dose-response data and create an updated or entirely new profile. Every step in generating CURATE.AI dose recommendations involved human oversight. Clinicians retained the discretion to accept, reject, or deem a recommendation irrelevant for a particular dosing event, for instance, when constrained by a strict drug administration schedule. Additionally, treatment response was monitored per RECIST criteria. The primary outcome of this study was defined as the percentage of participants in whom a CURATE.AI profile could be successfully applied, as determined by expert judgment based on numerical assessment of the primary outcome measure. Secondary and exploratory outcomes focused on the scientific and logistical feasibility of identifying key implementation factors, as well as on evaluations of treatment efficacy and toxicity. Ten patients were enrolled in palliative-intent treatment: single-agent capecitabine (1 patient), XELOX (6 patients), and XELIRI (3 patients). Disease monitoring was conducted using CEA in 9 patients and CA125 in 1 patient, with these biomarkers also serving as inputs to CURATE.AI. Four to five dose-response data pairs were sufficient to generate initial profiles. Among three responsive patients, the profiles enabled 11 CURATE.AI dose recommendations, all of which were accepted for prescribing and led to clinically meaningful dose adjustments. CURATE.AI is structured to ensure a central role for the physician in every dose-selection decision, and Blasiak et al. demonstrated physician adherence to 97.2% (35/36) of CURATE.AI’s recommendations (Blasiak et al., 2025).

## 5 Discussion

Across studies evaluating AI-based prediction of oxaliplatin response in CRC, some models consistently outperformed others depending on the types of data inputs used. In genomic-focused studies, Amniouel and Jafri (2023) demonstrated that RF consistently achieved the best results across validation and independent test cohorts, showing near-perfect accuracy, sensitivity, and specificity across all three regimens studied (FOLFOX, mFOLFOX, and mFOLFIRI). Similarly, Tsuji et al. (2012) validated RF superiority in an independent cohort reporting improvement in both sensitivity (81.4% vs. 91.3%) and specificity (85.1% vs. 95.6%) after outlier removal and achieving strong stratification of survival outcomes for responders and non-responders (2-year OS of 69.8% for responders and 38.6% for non-responders). Zhou et al. (2023) findings further reinforce the RF model’s reliability across other datasets, including transcriptomic datasets, particularly lncRNA datasets. In the study, four ML models (LASSO, DT, RF, and SVM) were used, yielding AUC values of 0.794–0.880.

SVM showed more variable performances. Lu et al. (2020) found that SVM achieved the best results with genomic data among the models studied (RF and neural network algorithms), with a sensitivity of 0.9% and a specificity of 0.69%. Additionally, in a cytokine-based prognostic classifier developed by van Timmeren et al. (2020), SVM combined with Cox proportional hazards modeling stratified patients into distinct risk groups; however, the accuracy, sensitivity, and specificity (0.749, 0.831, and 0.677) remained below RF benchmarks for genomic-based inputs. Similarly, Defeudis et al. (2021) reported only moderate predictive performance (as confirmed by independent validation) for a 3D approach using an SVM as the classifier (ACC ∼61%), especially in per-patient validation, where performance was poor. These results suggest that SVM may excel on moderately dimensional datasets (such as genomic data) with well-defined boundaries, but appear less potent when complex, heterogeneous cancer biology datasets are integrated (such as imaging and proteomic data).

Concerning imaging-based studies, Wei et al. (2021) demonstrated that ResNet10-based DL radiomics, when combined with CEA, significantly outperformed LASSO (AUC 0.745 vs. 0.598). CEA provided a slight boost to the DL-based model (AUC 0.489 vs. 0.558). These findings highlight DL’s ability to extract high-level spatial patterns from imaging data, even when handcrafted radiomics features often fall short. ML models yield conflicting outcomes when imaging data is integrated. For instance, Giannini et al. (2022) used a delta-radiomics DT and achieved AUCs of 0.99 in the training set and 0.93 in the validation set. However, as mentioned above, Defeudis et al. (2021) 3D per-lesion SVM performed moderately, and per-patient validation was poor. These findings suggest that DL may be a superior model for imaging-based prediction, as it outperformed one ML model in one study. In contrast, another ML model performed poorly in a different study.

Taken together, these findings indicate that no model is universally superior across all data types. This highlights the importance of data-modality-matched model selection. RF-based models seem superior for omics-based predictions, while DL-based models seem better for imaging-based predictions. Further research is needed to validate this superiority, and future studies should leverage these strengths by integrating omics, imaging, and clinical biomarker data to develop multimodal prediction systems.

## 6 Challenges and limitations

The molecular revolution in oncology continues to evolve, shifting from traditional pathology that relies on morphological and histological evaluation of tumor samples to a molecularly guided approach. In the AI era, precise individual molecular features are integrated into diagnosis, grading, and prognosis, enabling more personalized treatment strategies (Mondello et al., 2023). While AI holds immense potential in healthcare, numerous obstacles still hinder its implementation and widespread adoption. For instance, the rapid advancement of AI in healthcare has led to the emergence of tools and applications that often lack regulatory approval, raising both ethical and legal concerns. Hence, comprehensively examining and understanding the ethical and regulatory challenges associated with AI technologies in healthcare is vital to ensure responsible development and practical implementation (Mennella et al., 2024).

Given that AI models are trained on patients’ private and sensitive information, such as identity, health status, diagnoses, and other sensitive data, it is crucial to discuss the risks to data security. It is essential to emphasize that data collection without informed consent, data theft, or misuse violates patients’ rights and interests. Several methods exist to safeguard AI models and sensitive data from attacks. For example, homomorphic encryption is a method in which both the model and the data are encrypted, allowing only authorized parties to access them. This allows the model to be secured without compromising its performance. Unfortunately, applying this technique to larger networks remains impractical as the additional computational cost is enormous (Phong et al., 2018). Another method is differential privacy, which introduces noise during training to mask sensitive data, making it widely applicable but at the cost of reduced accuracy (Abadi et al., 2016). In generative approaches, a protected generative model leverages sensitive data to create synthetic data that an attacker cannot distinguish from the original. To achieve this, generative models such as generative adversarial networks (GANs) or variational autoencoders (VAEs) are trained using differential privacy. Models can then use the generated data without requiring additional protection measures (Creswell et al., 2018). Beyond these data-level protections, an increasingly adopted paradigm is federated learning, in which models are trained locally across institutions, and only model parameters are shared, enabling multi-center collaboration while keeping sensitive records on-site (Reddy et al., 2025). This approach has already been demonstrated in CRC, for instance in a multicenter federated model incorporating differential privacy to predict 90-day mortality following colorectal surgery (Yang and Yue, 2026). Even so, federated learning introduces its own limitations, including communication overhead, performance degradation due to heterogeneous data across institutions, and residual vulnerability to inference attacks on shared model updates (Reddy et al., 2025).

Having the algorithm developed and tested using data from a single institution makes its performance in other healthcare settings uncertain. This limitation is consistent with recent reviews of multimodal AI in CRC, which reported that many published models lack external validation and highlighted data heterogeneity as a major barrier to the generalizability and clinical translation of AI systems (Jonnagaddala et al., 2025). The problem is even more pronounced in the treatment-prediction setting most relevant to this review: a systematic review and meta-analysis of AI models predicting chemotherapy response and toxicity in metastatic CRC found that an external validation cohort was absent in more than half of the included studies (Russo et al., 2022). This variability in how the model performs across scenarios (such as patient subpopulations, geographic locations, and time) raises questions about algorithmic fairness, reliability, and the long-term safety of using prognostic AI models in oncology. Ensuring consistent model performance across patient subpopulations, such as those defined by race or gender, is crucial for achieving algorithmic fairness and an equitable distribution of interventions. However, risk prediction algorithms can unintentionally reflect and reinforce societal inequalities. As a result, these algorithms manifest as less accurate predictions for protected patient groups. Two main factors leading to biased models include the limited availability of training data for medically underrepresented populations and the use of inadequate proxy variables that poorly capture the actual underlying risk mechanisms (Kolla and Parikh, 2024; Obermeyer et al., 2019).

Another key challenge is the “black box” problem, which refers to the lack of transparency into how AI systems make decisions (Keike et al., 2018). For clinical use, it is almost mandatory that an AI can explain the reasoning behind its predictions. There are already a few promising AI applications for predicting treatment response that can provide such explanations and are therefore referred to as “white boxes” (Shimizu and Nakayama, 2020; Prelaj et al., 2022; Foersch et al., 2023). In practice, however, most current approaches rely on *post hoc* explainability methods such as SHAP (Shapley Additive Explanations), a game-theory-based technique that quantifies each input feature’s contribution to a model’s output, providing both global and local explanations through feature-importance rankings (Lundberg and Lee, 2017). In CRC, for example, SHAP has been used to link tumor heterogeneity features to pathological complete response in a model predicting neoadjuvant anti-PD-1 therapy response (Zhang et al., 2025).

Addressing liability relating to the use of AI is a particular challenge, mainly since clinical staff are accustomed to being responsible for their own decisions (Brady and Neri, 2020). The situation becomes more complex when AI guides, or even fully makes, clinical decisions, raising persistent questions relating to who or what is liable for a bad outcome. Potentially liable parties include the clinicians using the AI tool, the software developers who created it, the vendors who sell it, the healthcare organizations that implement it, or the regulators who approved it Liberati et al. (2017), Linthicum et al. (2019), Lai et al. (2020), Kelly et al. (2019), Ho et al. (2019). Traceability of harm is another concern. Due to the involvement of numerous parties, assigning responsibility becomes complicated, entailing both legal and moral dimensions. This diffusion of responsibility can lead to adverse outcomes, including the lack of compensation for individuals who have suffered harm, incomplete identification of the harm and its root causes, unaddressed damage, and a potential loss of public trust in these technologies when it seems that neither developers nor users can be held accountable (Mennella et al., 2024). This issue remains unresolved due to the complex interplay of legal and ethical concerns. Currently, healthcare professionals remain liable for their decisions, even when AI algorithms inform those decisions; they may not fully understand the implications (Ahmed et al., 2023).

## 7 Future prospects and concluding remarks

**FIGURE 6:**
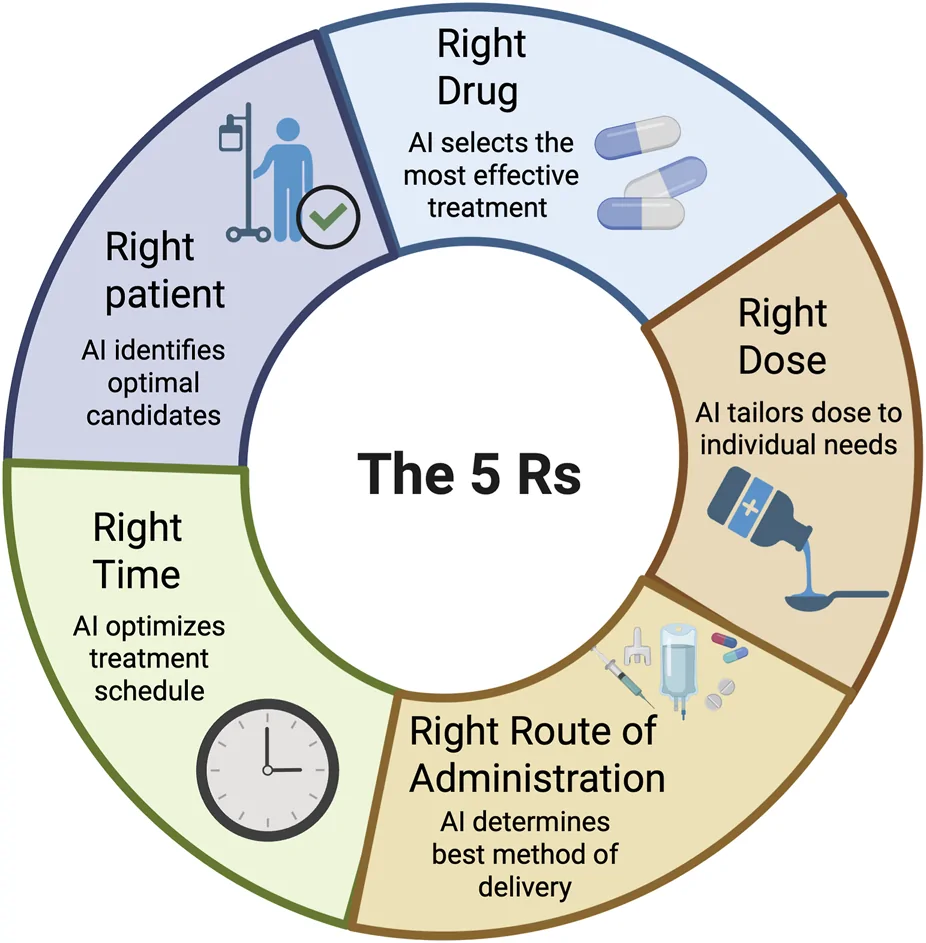
AI application in achieving the 5Rs: right patient, right drug, right dose, right route of administration, right time. Created in BioRender. El-Najjar, N. (2026) https://BioRender.com/v83z5xf.

As AI continues to evolve, its ability to support early decision-making, decrease late-stage drug development failures, and accelerate the creation of safer, more effective therapies becomes increasingly evident. AI enhances the traditional Five Rights of medication administration: the right patient, the right drug, the right dose, the right route, and the right time. As illustrated in Figure 6, AI facilitates the identification of the right patients through genomic, transcriptomic, proteomic, and radiomic data. Additionally, the right drug and dose can be selected by evaluating metabolic rate, renal/hepatic function, body composition, toxicity risk, and treatment tolerance. By evaluating pharmacokinetic parameters and delivery efficiency, AI can determine the most suitable route of administration. Lastly, it ensures the right timing by optimizing treatment schedules and preventing delays based on patient status and response patterns.

Despite those advancements, fully leveraging this potential requires a deeper understanding of AI’s real-world implications and limitations. Successful implementation relies not only on technological advances but also on a comprehensive awareness of pharmaceutical workflows, clinical practice, and regulatory requirements (Lee et al., 2025). Rigorous clinical trials are essential for validating AI-driven results and ensuring that developed models can be applied to broader patient populations. In this regard, it is vital that intelligent oncology be recognized as a new specialty and that more scientists, engineers, and clinicians be trained in its principles and applications (Lin et al., 2023). Through interdisciplinary collaboration and appropriate healthcare education, AI will increasingly serve as a cornerstone of modern oncology. The future of medicine is expected to be more accurate, efficient, and patient-centered, ushering in a new era in which intelligent oncology will play a vital role in detection and treatment.
