# Supplementary material for: Artificial intelligence applications in oxaliplatin-based chemotherapy for colon cancer: advancing prognosis, toxicity prediction, and dose personalization
Source: Front Pharmacol. 2026 Aug 10;17:1784272. doi: 10.3389/fphar.2026.1784272 (PMC13500912; doi:10.3389/fphar.2026.1784272)
Supplement: Supplementary file 1 [file Table1.docx]

**Supplementary Table 1.** Comparative summary of AI and machine learning studies reporting multi-omics biomarkers associated with oxaliplatin sensitivity and resistance in colorectal cancer

| Data Type | Classification Category | Data Expression / Findings | Reference |
| --- | --- | --- | --- |
| Genomic Data | Early-stage CRC | Genes relevant to drug response prediction: FOXA1, KRT23, GRM8, HOXA11, HOXA10, ABCB1, LEFTY1, CHRM3, OLMF4, LYZ | [1] |
| Genomic Data | Metastatic (late-stage) CRC | Genes relevant to drug response prediction: IFI44L, WIF1, LY6G6D, SFRP2, RETNLB, CMPK2, ACSL6, RSAD2, LOC100507477, IFIT1, MX1, HUNK |  |
| Genomic Data | Responders (FOLFOX) | Downregulated genes in responders: WASHC4, HELZ, ERN1, RPS6KB1, APPBP2 | [2] |
| Genomic Data | Responders (FOLFOX) | Upregulated genes in responders: IRF7, EML3, LYPLA2, DRAP1, RNH1, PKP3, TSPAN17, LSS, MLKL, PPP1R7, GCDH, C19ORF24, CCDC124 |  |
| Genomic Data | Resistance pathways (FOLFOX/bevacizumab or FOLFOX) | WNT signaling (BCL9, CDX2), EMT (INHBA, PRRX1, PBX1, YWHAE), chromatin remodeling (EP300, ARID1A, SMARC4, NSD3), DNA repair (WRN, BRIP1), NOTCH signaling (MAML2), cell-cycle regulation (CNTRL, CCNE1) | [3] |
| Genomic Data | Early-stage (disease-free) | Genes that identified disease-free patients: BRAF, FCGR2A, IGF1, MSH2, NAGK, NFE2L2, NQO1, PANK3, SLC47A1, SLCO1B1, UGT1A1 | [4] |
| Genomic Data | Predicted non-responders | SMURF2, MBTD1, AP3M2, RNF141, NPEPPS, BPTF, FAM73A, APPBP2, AMZ2P1, SRGAP1, NMT1, CSPP1, EIF1, CEP290 | [5] |
| Transcriptomic Data | Sensitivity markers | lncRNAs linked to oxaliplatin sensitivity: C20orf197, UCA1, MIR17HG, MIR22HG | [6] |
| Proteomic Data | Prognostic (OS correlation) | Cytokines correlated with overall survival: MDC, Flt3L, MCP-3, IL-8, IP-10, MCP-1 | [7] |

| Characteristic | All Patients (n=126) | Low SMI (n=59) | Without Low SMI (n=67) | p-value (for G3+) |
| --- | --- | --- | --- | --- |
| Incidence of Grade ≥3 Neutropenia | 28 (22%) | 18 (31%) | 10 (15%) | 0.036 |
| Incidence of Any Grade ≥3 Hematologic Toxicity | (Not specified directly for overall G3+ in the table, but 39% vs 24% in the text) | 39% | 24% | 0.067 |
| Median Time to Grade ≥3 Neutropenia | Not reached | Not reached | Not reached | 0.053 (HR 2.146) |
| 5-FU Bolus Relative Dose Intensity (RDI) | (Not specified for overall) | 52% | 65% | 0.021 |
| 5-FU Bolus RDI (Cycles 7-12) | (Not specified for overall) | 44% | 61% | 0.034 |
| Male Patients: Incidence of Grade ≥3 Hematologic Toxicity | (Not specified for overall) | 46% | 21% | 0.027 |
| Male Patients: Incidence of Grade ≥3 Neutropenia | (Not specified for overall) | 37% | 10% | 0.015 |

**Supplementary Table 2.** The relationship between low Skeletal Muscle Index (SMI) and grade ≥3 neutropenia in patients with metastatic colorectal cancer, who are treated with FOLFOX

SMI, Skeletal Muscle Index; LBM, Lean Body Mass; Vd, volume of distribution; TAT, Total Adipose Tissue; Cmax, peak concentration; GI, Gastrointestinal; RDI, Relative Dose Intensity; HR, Hazard Ratio; FOLFOX, Folinic acid (FOL), Fluorouracil (F), and Oxaliplatin (OX).

1.	Amniouel, S. and M.S. Jafri, *High-accuracy prediction of colorectal cancer chemotherapy efficacy using machine learning applied to gene expression data.* Front Physiol, 2023. **14**: p. 1272206.

2.	Lu, W., et al., *FOLFOX treatment response prediction in metastatic or recurrent colorectal cancer patients via machine learning algorithms.* Cancer Med, 2020. **9**(4): p. 1419-1429.

3.	Abraham, J.P., et al., *Clinical Validation of a Machine-learning-derived Signature Predictive of Outcomes from First-line Oxaliplatin-based Chemotherapy in Advanced Colorectal Cancer.* Clin Cancer Res, 2021. **27**(4): p. 1174-1183.

4.	Mucaki, E.J., et al., *Predicting responses to platin chemotherapy agents with biochemically-inspired machine learning.* Signal Transduct Target Ther, 2019. **4**: p. 1.

5.	Tsuji, S., et al., *Potential responders to FOLFOX therapy for colorectal cancer by Random Forests analysis.* Br J Cancer, 2012. **106**(1): p. 126-32.

6.	Zhou, Q.N., et al., *Oxaliplatin related lncRNAs prognostic models predict the prognosis of patients given oxaliplatin-based chemotherapy.* Cancer Cell Int, 2023. **23**(1): p. 103.

7.	Chen, Z.Y., et al., *A prognostic classifier consisting of 17 circulating cytokines is a novel predictor of overall survival for metastatic colorectal cancer patients.* Int J Cancer, 2015. **136**(3): p. 584-92.
